# Temporal Dynamics of Collective Resonances in Periodic Metasurfaces

**DOI:** 10.1021/acsphotonics.4c00412

**Published:** 2024-05-16

**Authors:** Radoslaw Kolkowski, Annemarie Berkhout, Sylvianne D. C. Roscam Abbing, Debapriya Pal, Christian D. Dieleman, Jaco J. Geuchies, Arjan J. Houtepen, Bruno Ehrler, A. Femius Koenderink

**Affiliations:** † Department of Applied Physics, Aalto University, P.O. Box 13500, Aalto FI-00076, Finland; ‡ Department of Physics of Information in Matter and Center for Nanophotonics, NWO-I Institute AMOLF, Science Park 104, Amsterdam 1098 XG, The Netherlands; § Advanced Research Center for Nanolithography (ARCNL), Science Park 106, Amsterdam 1098 XG, The Netherlands; ∥ Optoelectronic Materials Section, Faculty of Applied Sciences, Delft University of Technology, Van der Maasweg 9, Delft 2629 HZ, The Netherlands; ⊥ Institute of Physics, University of Amsterdam, Amsterdam 1098 XH, The Netherlands; # Department of Sustainable Energy Materials and Center for Nanophotonics, NWO-I Institute AMOLF, Science Park 104, Amsterdam 1098 XG, The Netherlands

**Keywords:** interferometric autocorrelation, two-photon excited luminescence, quantum dots, surface lattice resonances, quasi-BIC

## Abstract

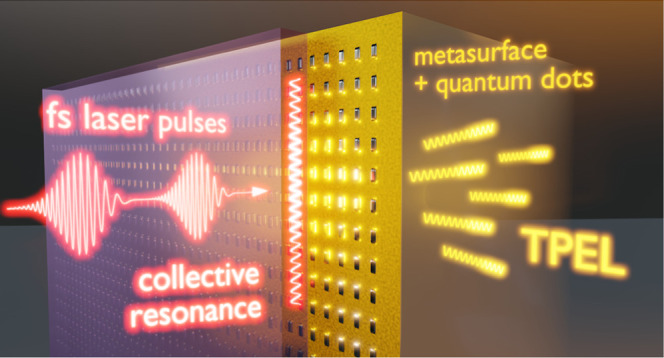

Temporal dynamics of confined optical fields can provide valuable insights into light–matter interactions in complex optical systems, going beyond their frequency-domain description. Here, we present a new experimental approach based on interferometric autocorrelation (IAC) that reveals the dynamics of optical near-fields enhanced by collective resonances in periodic metasurfaces. We focus on probing the resonances known as waveguide-plasmon polaritons, which are supported by plasmonic nanoparticle arrays coupled to a slab waveguide. To probe the resonant near-field enhancement, our IAC measurements make use of enhanced two-photon excited luminescence (TPEL) from semiconductor quantum dots deposited on the nanoparticle arrays. Thanks to the incoherent character of TPEL, the measurements are only sensitive to the fundamental optical fields and therefore can reveal clear signatures of their coherent temporal dynamics. In particular, we show that the excitation of a high-*Q* collective resonance gives rise to interference fringes at time delays as large as 500 fs, much greater than the incident pulse duration (150 fs). Based on these signatures, the basic characteristics of the resonances can be determined, including their *Q* factors, which are found to exceed 200. Furthermore, the measurements also reveal temporal beating between two different resonances, providing information on their frequencies and their relative contribution to the field enhancement. Finally, we present an approach to enhance the visibility of the resonances hidden in the IAC curves by converting them into spectrograms, which greatly facilitates the analysis and interpretation of the results. Our findings open up new perspectives on time-resolved studies of collective resonances in metasurfaces and other multiresonant systems.

## Introduction

Resonant optical systems are essential in many areas of modern science and technology. Since multimode resonant optical systems can exhibit complex temporal dynamics, studying their ultrafast optical response requires time-resolved methods. Examples of such systems include nanoparticle assemblies,^1−3^ nonlocal diffractive metasurfaces,^4,5^ anapoles,^6,7^ as well as recently explored time-varying systems.^8−10^ From the practical point of view, understanding and controlling the temporal dynamics can be crucial, e.g., for optimizing the efficiency of nonlinear optical processes and for tailoring their near- and far-field characteristics.^10−20^ Periodic metasurfaces are especially relevant in that context,^21−26^ as they can support collective resonances, in which Fano-like coupling between diffractive and localized resonances^27−32^ can make the temporal dynamics nontrivial. Among various types of collective resonances, including guided-mode resonances,^33−35^ surface lattice resonances (SLRs),^36−40^ and waveguide-plasmon polaritons (WPPs),^41−45^ of particular current interest are collective dark modes,^46−48^ in which radiation loss is suppressed by destructive interference. Currently, analogous to quantum mechanical systems,^49^ such modes are often called photonic bound states in the continuum (BICs).^50−52^ Surface roughness, finite size of metasurfaces, and asymmetric shape of their building blocks may convert BICs into quasi-BICs,^53−55^ allowing them to couple with external optical fields. Under illumination, quasi-BICs can show very high *Q* factors and large local field enhancement, which can be used for enhancing nonlinear frequency conversion and light emission.^56−58^ The research on collective resonances in periodic metasurfaces, including BICs and quasi-BICs, has so far mainly focused on their stationary spectroscopic properties, despite the fact that such resonances often explore various regimes of coupling, and therefore may exhibit very interesting temporal dynamics, such as Rabi oscillations and Fano-like interference.^1,2,59−64^

Having established the large need to quantify temporal near-field dynamics in nanophotonic systems, the question is how to do so. One of the simplest time-resolved optical methods to map the dynamics of optical fields is interferometric autocorrelation (IAC),^65−67^ in which nonlinear emission is measured as a function of time delay between two identical optical pulses. IAC and its spectrally resolved versions, e.g., frequency-resolved optical gating (FROG) and interferometric frequency-resolved autocorrelation, are widely used to characterize ultrashort laser pulses.^68−75^ They have also been used to reveal the temporal evolution of optical fields at the nanoscale,^76−78^ with particular focus on studying the dephasing times in plasmonic nanoparticles^79−84^ and waveguide-coupled metal nanostructures.^27,31,85,86^ Adding spatial resolution to these techniques allows one to investigate the dynamics of various optical phenomena, such as spatiotemporal light localization,^87^ plasmonic hot spots,^88−90^ strong light–matter coupling,^91^ and surface plasmon polaritons.^92,93^ IAC usually generates its signal from coherent nonlinear effects, such as second- and third-harmonic generation (SHG and THG). In such coherent approaches, the measurements can provide spectral information about the incident (fundamental) optical fields, as this information is imprinted on the spectrum of the emitted (harmonic) optical fields.^27,73,76,83,86,91,93^ On the other hand, in the case of an incoherent nonlinear process such as two-photon-excited luminescence (TPEL), the spectroscopic information is lost.^73^ Nevertheless, the coherence of the fundamental fields enables one to study and control the dynamics of light using incoherent nonlinear effects such as TPEL.^15−18^ Indeed, TPEL has been considered as an alternative to SHG for pulse autocorrelation measurements.^94^ Two-photon absorption is another example of an incoherent nonlinear process which has been successfully employed to study the temporal dynamics of optical fields.^95^ One of the most advanced methods utilizing an incoherent nonlinear process is two-photon photoemission electron microscopy, which provides state-of-the-art spatial and temporal resolution for the investigation of nanoscale optical fields.^96−100^

**Figure 1:**
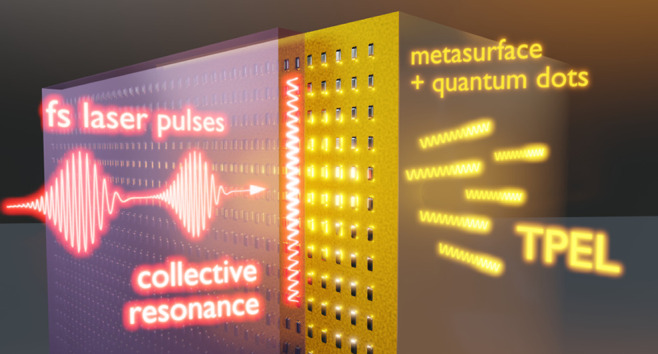
Illustration of the studied system. Plasmonic nanoparticle array (metasurface) is covered by a thin layer of QDs and embedded in a slab waveguide. Incident laser pulses excite high-*Q* collective resonances, which in turn enhance the local optical fields, giving rise to enhanced TPEL from QDs.

In this work, we develop a TPEL-based IAC technique to study the temporal dynamics of optical fields due to collective resonances in periodic metasurfaces. For studying collective resonances in periodic metasurfaces, it is highly desirable to have a technique in which the IAC signal is efficiently generated through the near-field enhancement, while still operating using a spatially uniform plane-wave illumination. This is because collective resonances usually exist at the band edges of photonic modes, which renders their momentum–space distribution extremely narrow. The approach that we demonstrate here perfectly fits the above requirements. Our samples are made of arrays of silver nanoparticles covered by a thin layer of CdSe/CdS/ZnS core/shell/shell quantum dots (QDs) and embedded in a dielectric slab waveguide (see Figure 1). The QDs used in our experiments exhibit highly efficient TPEL^101^ under irradiation by near-infrared femtosecond pulses. The nanoparticle arrays at hand exhibit high-*Q* resonances due to hybridization with the guided modes of the waveguide. Numerical simulations show that these resonances can be regarded as quasi-BICs, as they exhibit strong multipolar character and symmetry-dependent excitation efficiency.^54,55,102^ During the experiments, the incident pulses excite the high-*Q* resonances, which enhance the TPEL emission from QDs. The stability and brightness of the enhanced TPEL are sufficient for wide-field imaging on a camera, allowing us to simultaneously study many arrays of different geometrical parameters. The large advantage of TPEL as an incoherent process for IAC, apart from the superior brightness and stability over, for instance, SHG from our sample, is that the IAC signal is insensitive to coherent interference effects at the emission frequency (such as the influence of its polarization and phase). Thereby, we strictly probe only the dynamics of optical fields at the fundamental frequency.

To summarize the findings of this paper and explain its organization, first, in the section Experimental Approach, we report on the experimental procedures to measure IAC by TPEL, and we provide a formalism expressing the TPEL IAC in the fundamental input field. We study the IAC as a function of metasurface geometry (section Results and Discussion) and find that the measured IAC curves depend strongly on the shape of the nanoparticles and the lattice period. Particularly in metasurfaces supporting a single high-*Q* resonance near the excitation frequency, the IAC curves contain additional temporal interference fringes that reveal coherent oscillations at a single frequency. Remarkably, the fringes are clearly visible even when the incident pulses (each having a duration of 150 fs) are delayed from each other by > 500 fs. Such long-lasting oscillations indicate a high *Q* factor of the excited resonance. On the other hand, simultaneous excitation of two high-*Q* resonances in metasurfaces containing larger nanoparticles gives rise to a beat pattern, resulting from the interference of oscillations at two different frequencies. The observed signatures of temporal dynamics are well reproduced by a simple analytical model that we report on in section “IAC Analysis”. The analysis and interpretation of the results are further supported by FROG-like spectrograms created by Fourier-transforming the IAC curves and deconvolving the obtained spectra with reference. Creation of such spectrograms is enabled by the high quality of the measured IAC curves, which can be attributed not only to the brightness and stability of the TPEL signal but also to the high interferometric stability of the birefringent delay line^67,74^ used in our IAC measurements. Fitting the data with the proposed model allows us to estimate the frequencies of the collective resonances, their *Q* factors, and their relative contributions to the TPEL emission enhancement. The *Q* factors are found to be in the range 50–225. Although these values are modest compared to the state-of-the-art optical resonators, they can still be considered as relatively high for plasmonic systems,^103,104^ which is why we refer to the studied resonances as “high-*Q*” throughout this paper. Our results show that the excitations of high-*Q* collective resonances in periodic metasurfaces can exhibit nontrivial temporal dynamics, which can be revealed by the IAC measurements based on the enhanced TPEL emission. We believe that using QDs as the near-field nonlinear optical nanoprobes^105^ is an efficient and feasible approach with significant potential for future time-resolved characterization of resonant optical systems.

## Experimental Approach

### Metasurface Structure and Composition

**Figure 2:**
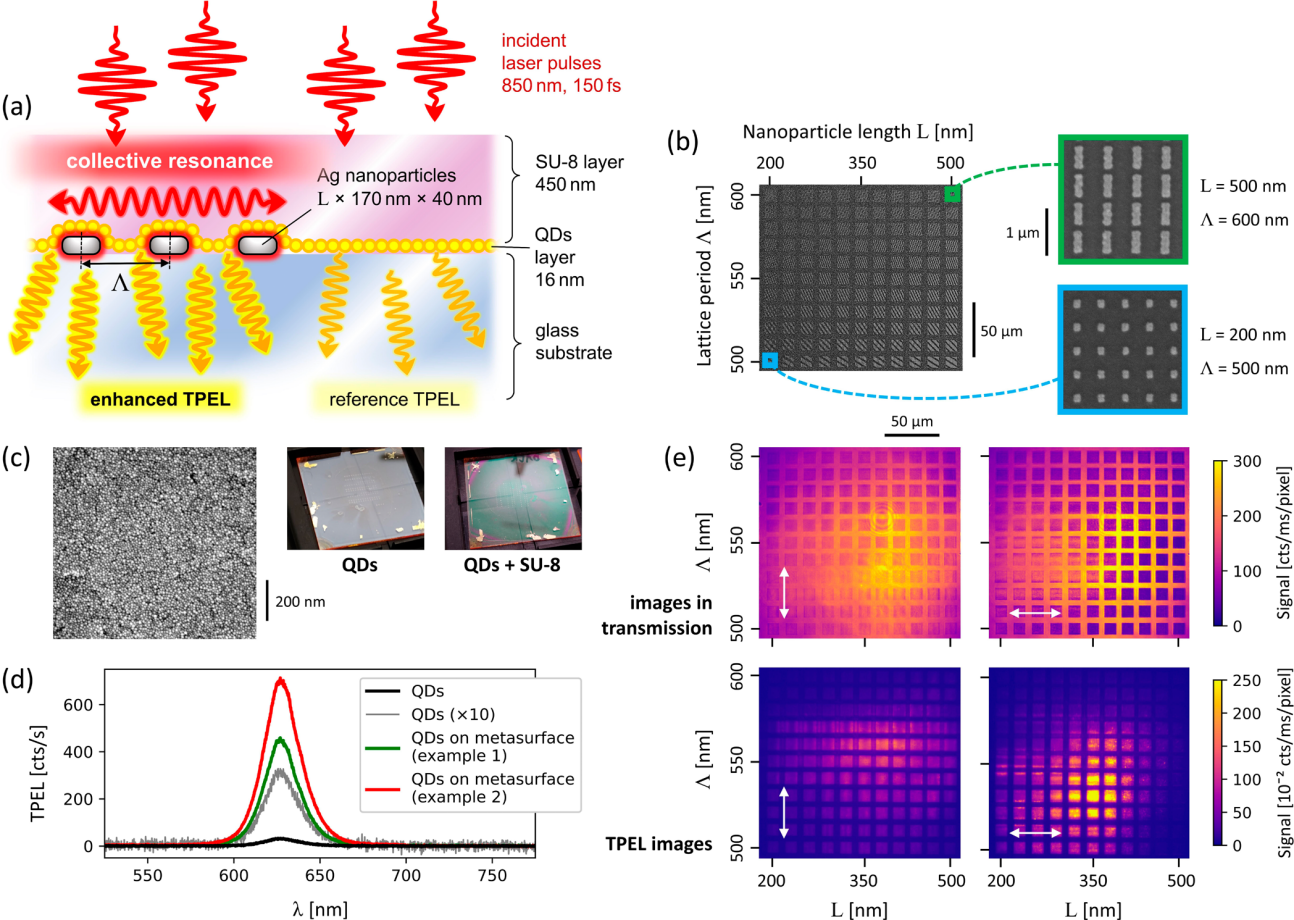
(a) Structure of the studied samples. (b) SEM images of the samples before depositing QDs and SU-8; on the left—11 × 11 = 121 nanoparticle arrays under investigation, each with different lattice period Λ and nanoparticle length L; on the right—close-ups of the arrays with extreme parameters. (c) Left—example SEM image of the QDs deposited by spin-coating, forming a continuous thin layer on a substrate; middle and right—photographs of the sample after spin-coating QDs and SU-8, respectively. (d) TPEL emission spectra; black—QD layer without enhancement (reference TPEL); gray—the same data multiplied by 10; green and red—examples of TPEL emission enhanced by metasurfaces of different parameters; green (example 1)—Λ = 560 nm, L = 350 nm, illuminated by incident light linearly polarized along the long axis of the nanoparticles; red (example 2)—Λ = 540 nm, L = 350 nm, for the incident light of opposite polarization. (e) Images of the arrays under different incident polarizations (as indicated by white arrows), based on the transmitted light (top row) and TPEL signal (bottom row). The examples presented in (d) are chosen based on the brightest regions of the TPEL images in (e).

Figure 2a shows the structure of the studied samples. We study metasurfaces consisting of silver nanoparticles of height 40 nm, width 170 nm, and various lengths L in the range from 200 to 500 nm (changed in steps of 30 nm). The nanoparticle arrays were fabricated using electron beam lithography, following the same procedure as described in refs (106–108). Example scanning electron microscopy (SEM) images of the arrays obtained directly after lithography are presented in Figure 2b. The size and shape of metal nanoparticles determine the properties of their localized surface plasmon resonances (LSPR).^109^ The relatively large size of the nanoparticles used in our metasurfaces allows them to support higher-order multipolar LSPR modes,^28,110^ and the variation of L makes it possible to tune their resonance frequencies and their contribution to the collective resonances. The nanoparticles are arranged into square lattices of various periods Λ in the range from 500 to 600 nm (changed in steps of 10 nm). Since Λ determines the diffractive resonance condition, tuning both L and Λ allows us to explore collective resonances that exhibit various degrees of hybridization while spectrally overlapping with the incident light frequency. The arrays are covered by a 16 nm thick layer of CdSe/CdS/ZnS core/shell/shell QDs that generate the TPEL signal and subsequently by a 450 nm thick layer of SU-8 polymer (see Figure 2c). At the fundamental wavelength (λ = 850 nm), the refractive index of the polymer (Microchem SU-8 2000, *n* = 1.575) is higher than the refractive index of the substrate glass (Schott D263, *n* = 1.515), so that the polymer layer supports the fundamental TE- and TM-guided modes. Multiple scattering of these modes by the nanoparticles gives rise to the collective resonances. Throughout the literature, such resonances are most often called WPPs,^41−45^ although their hybrid character makes them similar to SLRs.^36−39^ WPPs and SLRs differ in the degree of confinement of optical waves that mediate the coupling between the local nanoparticle resonances. Recently, WPPs and SLRs, as well as their nonplasmonic counterparts (guided-mode resonances, Bragg resonances)^33−35^ are often referred to as quasi-BICs^52,57^ whenever their *Q* factors are enhanced by the reduced radiative damping. Picking one of the above names (WPPs, SLRs, or quasi-BICs) would overemphasize only one distinctive feature of the studied resonances. Therefore, we decided to call them “collective resonances” throughout this article.

### TPEL Enhancement

At the heart of our method are wurtzite CdSe/CdS/ZnS core/shell/shell QDs that generate bright TPEL signals. The QDs are synthesized using the hot injection method, as described in refs (111 and 112,) yielding a colloidal solution in which the QDs were capped with a mixture of oleate, oleylamine, thiolate, phosphate, and phosphonate-stabilizing ligands. This allows them to be deposited in the form of a continuous thin layer (see Figure 2c) on the substrate and nanoparticles. These QDs show bright TPEL when pumped at wavelengths between 790 and 910 nm. The spectrum of the TPEL emitted by QDs consists of a single peak with the central wavelength 627 nm and spectral width 23 nm [full width at half-maximum (fwhm), see Figure 2d]. Despite containing noncentrosymmetric materials (CdSe, CdS, and ZnS), the QDs do not emit any detectable second-harmonic generation (see Figure S1 in the Supporting Information), which can be explained by their small volume and the destructive interference of coherent radiation from many randomly oriented SHG dipoles (the orientation of which depends on the local crystal lattice orientation).^113,114^ In our case, the nonlinear emission from QDs is spectrally separated from the potential SHG, allowing us to unambiguously recognize it as TPEL. However, in many materials, SHG and TPEL can be present simultaneously and overlap spectrally with each other, creating a challenge for ultrafast spectroscopy. In such cases, the two emission channels can be distinguished on the basis of their coherence properties through spectrally resolved autocorrelation measurements.^73^

Resonant enhancement of the local near-fields around the nanoparticles leads to >10-fold enhancement of the measured TPEL (compared to TPEL from bare QDs) without changing the emission spectrum, as can be seen in Figure 2d. The unchanged spectrum allows us to assume that the TPEL enhancement originates primarily from the local field enhancement at the fundamental frequency and not from the directional enhancement by a diffractive resonance at the emission frequency. Although the latter can be spectrally blurred by collecting the signal over a wide range of angles within the numerical aperture of the objective (in our case: NA = 0.7), the strongest enhancement usually occurs within a narrow spectral range near a band edge, and its spectral line shape is often highly asymmetric. The enhanced TPEL spectra in Figure 2d show no sharp features and no asymmetry, which suggests that the directional enhancement is insignificant. The unchanged spectrum also confirms that the enhanced TPEL emission comes from the QDs, and not from the metal nanoparticles, which are also known to be capable of producing various types of nonlinear emission.^12,115−117^ Our studies have shown that, under similar excitation conditions, the QDs’ TPEL is roughly by 1 order of magnitude brighter than that of rhodamine 6G (when used as a SU-8 dopant) and by 3 orders of magnitude brighter than the nonlinear signal emitted by metal nanoparticles. Moreover, the TPEL intensity depends quadratically on the input intensity over a large range of intensities (see Figure S2 in the Supporting Information), in contrast to the dim nonlinear emission of metal nanoparticles alone that is polluted by, e.g., thermal emission effects.

The QDs’ TPEL is so bright and stable that it offers the unique opportunity for wide-field imaging of multiple metasurfaces simultaneously and with a relatively short exposure time. As a result, the IAC measurements performed in this way have unprecedented resolution and signal-to-noise ratio compared to other plasmonic IAC experiments based on intrinsic nonlinear emission from metal nanostructures. The bottom row of Figure 2e shows two examples of TPEL images obtained during the measurements (in this case, the exposure time was 100 ms). The imaged area corresponds directly to that presented in the SEM image in Figure 2b. The TPEL images can also be directly compared with the transmission images in the top row of Figure 2e.

### Interferometric Autocorrelation

**Figure 3:**
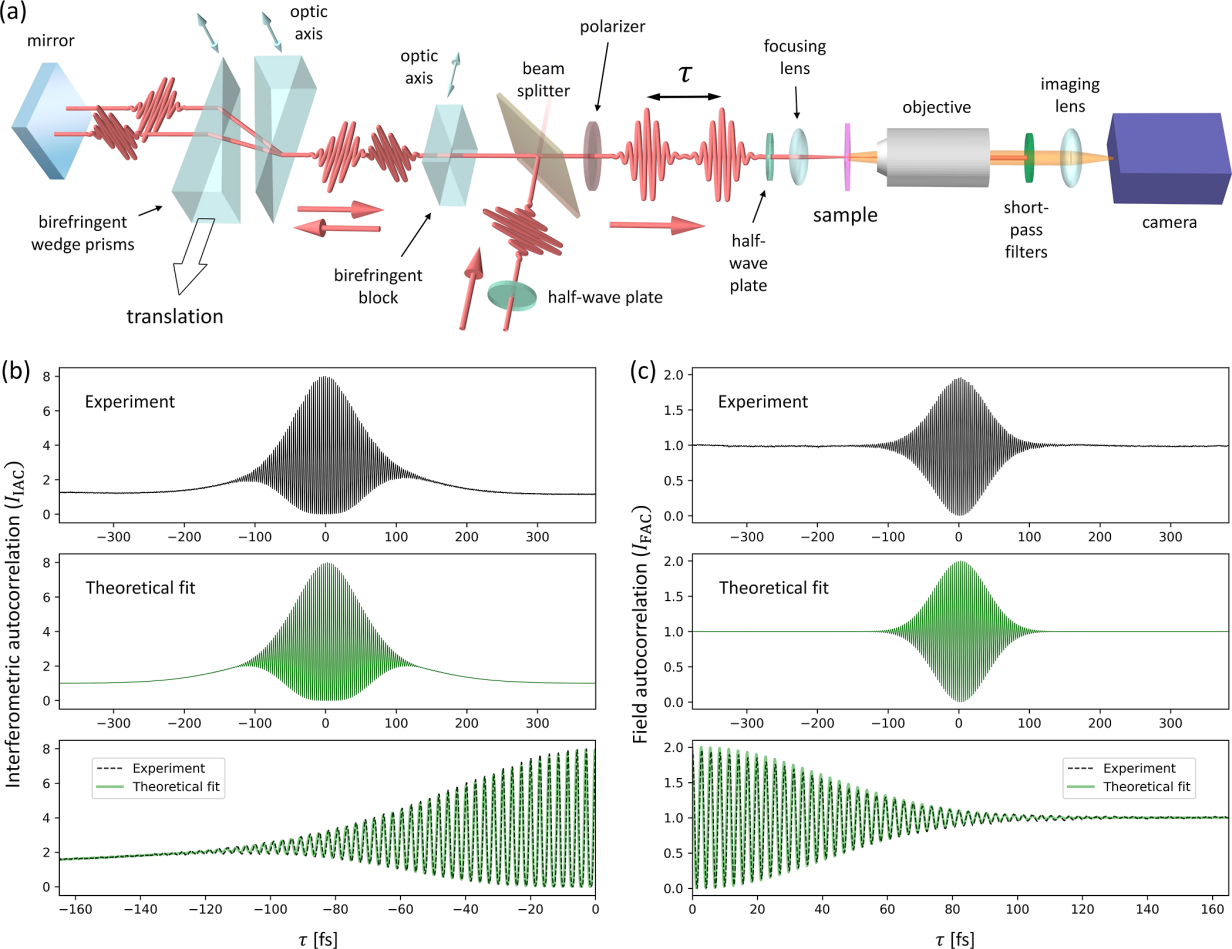
(a) Simplified scheme of the experimental setup. Description and technical details are provided in the “Methods” section and in Figure S3 in the Supporting Information. (b) Black—example reference IAC curve obtained by measuring TPEL from QDs without enhancement; green—theoretical fit based on eqs 1 and 3: pulse duration *T* = 150 fs, chirp *C* = 5.5 × 10^–5^ rad/fs^2^. (c) Black—field autocorrelation obtained by measuring the transmitted light; green—theoretical curve, obtained using eqs 3 and 4, assuming the same parameters as in (b). The plots on the bottom of (b,c) are the close-up views of the upper plots and show the good correspondence between the theoretical and experimental curves.

Figure 3a shows a simplified scheme of the setup that we use for IAC imaging of metasurfaces. A first main characteristic of the setup is that it is an entirely common-path microscope, in which we create pulse pairs with controllable delay using a birefringent delay line based on the design by Brida et al.^67^ The advantage of the common-path layout is that if sample and reference signal are both measured from the microscope object plane, any changes in IAC can be strictly attributed to the sample dynamics. The second main unique characteristic of the setup is that the IAC measurements are performed by recording the TPEL signal with a CCD camera as a function of the time delay τ between the incident pulses, as opposed to using a bucket detector. This is possible because the TPEL is very bright, and has the advantage that the IAC can be spatially resolved over the entire field of view. We use this to study many metasurface fields in parallel. Further details of the setup operation are provided in the “Methods” section.

Since QDs are deposited everywhere on the glass substrate, we can easily obtain a reference TPEL signal (without the resonant enhancement) by shifting the nanoparticle arrays outside the field of view of the camera. An example IAC curve obtained in this way is presented in Figure 3b. We use it to determine the basic parameters of the incident pulses by fitting the experimental data (black line) with a theoretical curve (green line) based on the formula
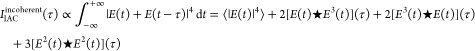
1where “⟨···⟩” is the average over *t*, “[.★.](τ)” stands for the cross-correlation, and the field *E*(*t*) is real-valued. This formula works out to be nearly identical to that for an IAC curve based on a coherent quadratic process such as SHG (note the crucial definition difference between |.|^4^ and |[.]^2^|^2^)
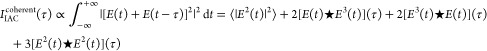
2

In the case of SHG, the first term, , depends on the generated field at the second-harmonic frequency, *E*^2^(*t*), which makes it sensitive to local field effects and, e.g., phase matching effects, at this frequency. In contrast, in the case of TPEL, the first term, ⟨|*E*(*t*)|^4^⟩, depends only on the incident field intensity, |*E*(*t*)|^2^. In addition, although the *E*^2^(*t*) terms appear in both equations, they correspond directly to the emitted field only in the case of a coherent process. This in fact makes an incoherent process such as TPEL superior for studying the coherent temporal dynamics of *E*(*t*), as it removes the effects of such dynamics, including the interference effects, at the emitted frequency. For both coherent and incoherent IAC, the first term is constant with respect to τ. Therefore, we use it to normalize the IAC curve by setting ⟨|*E*(*t*)|^4^⟩ ≡ 1. The resulting normalized IAC attains values in the range from 0 to 8. The peak-to-background ratio of 8 is consistent with TPEL exhibiting a quadratic power dependence (just like SHG), which is further confirmed in Figure S2 in the Supporting Information. In addition, Figure S1 shows that, contrary to SHG, the emitted TPEL spectrum does not depend on the fundamental wavelength, as expected from an incoherent spontaneous emission process.

For fitting our reference IAC measurements we assume that *E*(*t*) entering eq 1 is a chirped Gaussian pulse
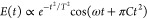
3

The fitted parameters are the pulse duration *T* and the chirp *C*, and their values are found to be 150 fs and 5.5 × 10^–5^ rad/fs^2^, respectively. The chirp is responsible for the fringe-less “shoulders” sticking above the flat background on both sides of the interference fringes in the central part of the IAC curve. It is noteworthy that the experimental data presented in Figure 3b are of exceptionally high quality, despite TPEL being measured from a very thin layer of bare QDs without the resonant metasurface enhancement. In addition to the excellent brightness and stability of TPEL from QDs that boosts the signal-to-noise ratio, the high quality of the obtained experimental data can also be attributed to the unique interferometric stability^74^ of the common-path design of the birefringent delay line used in our measurements. The high signal stability allows us to perform the measurements with a high τ resolution of around 0.1 fs, corresponding to around 25 data points per fringe, which greatly increases the confidence of analysis and interpretation of the measured IAC lineshapes.

Measuring the transmitted light instead of TPEL allows us to easily obtain another reference—the field autocorrelation (FAC). An example experimental FAC curve is presented in Figure 3c (black line). Contrary to IAC, FAC is based on linear interference, which makes it insensitive to the spectral phase. As a result, the FAC curve does not show a clear signature of the chirp. Instead, the chirp causes the pulse to appear shorter than it actually is. The FAC theoretical curve is based on the formula
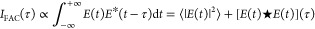
4

where *E*(*t*) and *E*(*t* – *τ*) in the integrand are complex-valued. The normalization is done by setting ⟨|*E*(*t*)|^2^⟩ ≡ 1. The values of the normalized FAC are in the range from 0 to 2. The theoretical curve, shown in Figure 3c (green line), is obtained using the parameters that were fitted to the IAC curve in Figure 3b. There is a good quantitative agreement between the theoretical and experimental curves for both IAC and FAC. This indicates that the simple formula in eq 3 is sufficient to represent the incident pulses, and that the obtained values of the fitted parameters *T* and *C* can be considered as accurate.

Figure 3c illustrates the fundamental limitation of linear interferometry in studying ultrafast optical phenomena, justifying the need for nonlinear effects such as TPEL. In particular, it shows that TPEL cannot be simply replaced by one-photon fluorescence (using a shorter wavelength to excite the samples), because doing so would make it impossible to correctly determine the incident pulse duration from the reference measurements. On the other hand, one-photon fluorescence could be used in cases where precise referencing is not needed, e.g., in resonant systems with very high *Q* factors, in which the incident pulse duration is negligible compared to the photon lifetime.

## Results and Discussion

### Experimental Signatures of a High-Q Resonance

**Figure 4:**
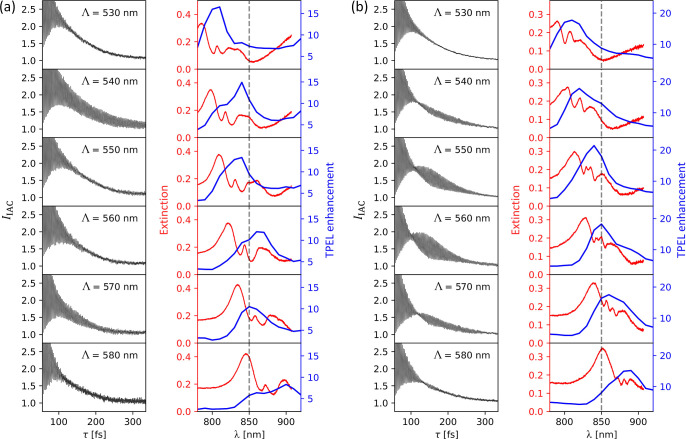
Resonant behavior of the metasurfaces made of nanoparticles with length *L* = 200 (a) and *L* = 350 nm (b) as a function of the lattice period Λ (indicated in the plots); columns on the left: close-ups of the measured IAC curves (*I*_IAC_, black curves); columns on the right: extinction spectra (red curves) and TPEL enhancement spectra (blue curves). The wavelength λ = 850 nm at which the IAC measurements are performed is marked by the dashed vertical line. The incident light was linearly polarized along the long axes of the nanoparticles, which corresponds to the vertical polarization in Figure 2e (the results for the opposite polarization are shown in the Supporting Information in Figure S4).

Figure 4 shows experimental data obtained for resonant metasurfaces composed of silver nanoparticles of length *L* = 200 nm (Figure 4a) and *L* = 350 nm (Figure 4b), with lattice period Λ varying from 530 nm (top rows) to 580 nm (bottom rows). All the results presented in Figure 4 were obtained for incident light polarized along the long axis of the nanoparticles (the results for the opposite polarization are presented in the Supporting Information, Figure S4). The close-ups of the measured IAC curves (black curves in the left columns in Figure 4a,b) are supplemented by corresponding extinction and TPEL enhancement spectra (red and blue curves in the right columns). The experimental details are provided in the “Methods” section and in the Supporting Information, Figure S3.

The IAC curve obtained for the lattice period Λ = 540 nm in Figure 4a (nanoparticles with *L* = 200 nm) contains strong interference fringes at τ > 250 fs, which are absent in the reference IAC curve (see Figure 3b). The additional interference fringes are due to the resonantly enhanced optical field that continues to oscillate after the incident pulse is gone.^118^ This allows the local field to interfere with the field excited by the second incoming pulse, even if it arrives after a significant time delay. Similar effects have previously been observed in the IAC measurements of other resonant systems.^7,27,31,79−82,84−86,90,91,93^ However, most of the previous studies focused on relatively low-*Q* plasmonic resonances with oscillations decaying within a few tens of femtoseconds, requiring much shorter pulses to reveal the resonant behavior. In our case, the fringes are visible at much larger delays due to the contribution of high-*Q* resonances, and therefore can be observed using relatively long pulses (150 fs).

The IAC fringe patterns are strongly dependent on the metasurface parameters and excitation conditions. For example, the additional fringes disappear at Λ differing by as little as 10 nm (Λ = 530 nm and Λ = 550 nm), while reappearing at Λ = 570 nm. The fringes are also significantly less pronounced under the opposite polarization of incident light (see Figure S4a in the Supporting Information). This indicates that the observed resonance is a collective resonance resulting from the hybridization between an LSPR (polarization dependence) and a diffractive guided mode resonance (dependence on Λ). In the case of a weak hybridization, the resonance wavelength λ_res_ is approximately determined by the lattice Bragg condition λ_res_ = Λ*n*_eff_, where *n*_eff_ is the effective refractive index of the guided mode. Based on the observed dependence, we can estimate that *n*_eff_ ≈ 850/540 ≈ 1.574, which is a reasonable value for an SU-8 slab waveguide (*n* = 1.575) on a Schott D263 glass substrate (*n* = 1.515). This interpretation in which clear pulse elongation occurs near the Bragg condition is consistent with the extinction and TPEL enhancement spectra presented in the right column in Figure 4a (red and blue curves). Both types of spectra show resonant peaks that are red-shifted as Λ is increased, as expected from the Bragg condition. The extinction spectra reveal several resonant peaks, in contrast to the TPEL enhancement spectra which do not show so many features. This results from the fact that the TPEL enhancement spectra are convolved with the bandwidth of the incident laser pulses (fwhm ≈ 20 nm). Moreover, TPEL is a probe of the local field enhancement, which can be stronger at a high-*Q* resonance that has a relatively small extinction. The resonant interference fringes in the IAC curve for Λ = 540 nm are probably caused by such a high-*Q* resonance that is not very pronounced in the extinction spectrum, but gives rise to a significant TPEL enhancement.

### Numerical Simulations Revealing Multipolar Quasi-BICs

**Figure 5:**
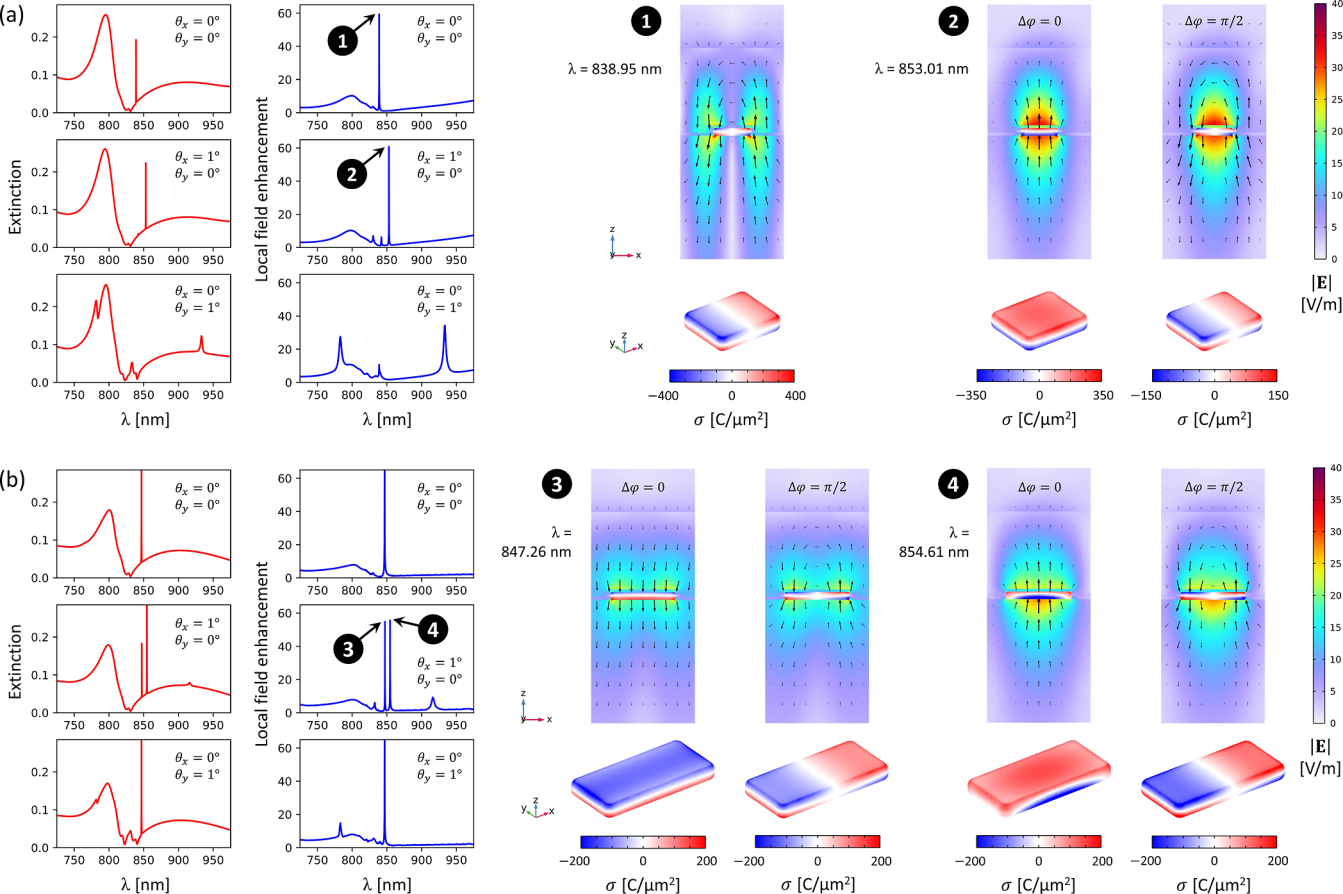
Numerical simulations (using COMSOL Multiphysics, Wave Optics module) of the high-*Q* collective resonance excitations in metasurfaces with nanoparticle length *L* = 200 (a) and 350 nm (b); red curves—extinction spectra, blue curves—spectral dependence of the maximum local field enhancement, 2D plots in the *xz*-plane—electric field norm |**E**| (color) and instantaneous electric field vector distribution (black arrows) over one unit cell of the metasurface, 3D plots—instantaneous surface charge density σ on the nanoparticle surface. The incident field is polarized in the *xz*-plane (mainly along *x*) and has an amplitude of 1 V/m. The extinction and field enhancement plots are obtained under various incidence angles θ_*x*_ (in the *xz*-plane) and θ_*y*_ (in the *yz*-plane), as indicated, while the 2D and 3D plots show the results at various oscillation phases Δφ, as indicated (except the resonance marked by number 1, which shows only one type of oscillation). In both (a,b), we assumed lattice period Λ = 550 nm, nanoparticle width = 170 nm, nanoparticle height = 40 nm, QD layer thickness = 15 nm, QD layer refractive index = 2 (based on ellipsometry measurements), waveguide thickness = 450 nm (including SU-8 and QDs). Optical constants of the glass substrate, silver, and SU-8 were taken from the refractiveindex.info database.^119^

To investigate the origin and character of the high-*Q* resonances revealed by the IAC curves, we conducted numerical simulations in COMSOL Multiphysics, including calculations of the extinction, local field enhancement, surface charge distribution, and multipole expansion. The calculated extinction and field enhancement spectra (for Λ = 550 nm, *L* = 200 nm) are presented in Figure 5a, together with spatial distributions of the electric field (**E**) and surface charge density (σ) at selected resonance peaks. In order to approximate the effects of the symmetry breaking caused by possible experimental misalignment, fabrication imperfections, finite numerical aperture, and finite size of the metasurfaces, we have introduced in the simulation a nonzero angle of incidence, either in the *xz*-plane (θ_*x*_) or in the *yz*-plane (θ_*y*_).

The calculated spectra in Figure 5a show qualitative agreement with the measured extinction spectra in Figure 4a, except for the presence of ultranarrow peaks, which were not captured by the measurements. Based on the spectral width of these peaks, the *Q* factors can be estimated as *Q* ≈ 1.1 × 10^4^ (peak number 1) and 1.4 × 10^4^ (peak number 2). These values are significantly higher than those reported for SLR-based plasmonic metasurfaces.^40^ This is not surprising, taking into account recent theoretical predictions for WPPs showing that their *Q* factors do not actually have an upper limit.^44^ The high-*Q* resonance marked by number 1 can be excited even at normal incidence, without breaking the symmetry. The surface charge density shows that this resonance is a dark mode resulting from the hybridization of an electric quadrupole LSPR with a TM-polarized guided-mode resonance having a node at the nanoparticle position. This multipolar excitation is allowed by the relatively large size of the nanoparticles (200 × 170 × 40 nm). Interestingly, at θ_*x*_ = 1°, this resonance is no longer efficiently excited. Instead, a second peak emerges (number 2), corresponding to a strong electric dipole along *z*. This resonance is associated with the hybridization of the LSPR with a TM-polarized guided mode resonance having an antinode at the nanoparticle position. At normal incidence, this peak is a symmetry-protected BIC, but it becomes a quasi-BIC at oblique incidence.^55^ The multipole expansion of this excitation, based on the formulas in ref (120) is presented in Figure S5 in the Supporting Information. It should be pointed out that our numerical model neglects significant surface roughness of the nanoparticles (see Figure 2b) which will contribute to the symmetry breaking and increase the excitation efficiency and radiative loss of the BIC and quasi-BIC resonances.^53^

### Multiresonant Response

Figure 4b shows the results analogous to Figure 4a but for even longer nanoparticles (*L* = 350 nm). Here, the IAC curves (black curves/left column) reveal interference fringes that form a beat pattern. The pattern depends on the lattice period Λ, reaching maximum visibility at Λ = 560 nm and disappearing at Λ = 530 and 580 nm. The pattern is also polarization-dependent, vanishing under the opposite polarization (see Figure S4b in the Supporting Information). Similar beating has been reported in several previous IAC studies,^7,27,80,85,86,91^ as well as predicted by several theoretical works.^1,2,6^ A likely explanation is simultaneous excitation of two resonances, leading to interference of two different frequency components. Extinction spectra (red curves in the right column in Figure 4b) indeed show two closely spaced peaks (increasingly apparent as Λ exceeds 540 nm), suggesting a contribution of another resonance compared to metasurfaces with smaller nanoparticles in Figure 4a. In the TPEL enhancement spectra (blue curves in Figure 4b), the individual peaks of the closely spaced resonances are not resolved. Instead, the spectra show a single TPEL enhancement peak that red-shifts with increasing Λ. For Λ = 560 nm, both the double peak in the extinction spectrum and the single peak in the TPEL spectrum coincide with the fundamental wavelength (λ = 850 nm). This coincidence further supports the explanation that it is the interference between two high-*Q* resonances of slightly different frequencies that causes the beat pattern to appear in the measured IAC curves. Numerical simulations presented in Figure 5b (analogous to Figure 5a) show that at θ_*x*_ = 1°, two different resonances (marked by numbers 3 and 4, with *Q* ≈ 1.1 × 10^4^ and 0.8 × 10^4^, respectively) can be excited simultaneously and with comparable magnitudes. This is in contrast to the case presented in Figure 5a, where the spectra are dominated by only one peak at a time. The electric field and surface charge density show that each of the two resonances in Figure 5b is a mixture of an electric dipole along *z* and an electric quadrupole. The multipole expansion presented in Figure S6 in the Supporting Information shows a significant contribution of higher-order multipoles (especially electric quadrupoles and magnetic dipoles) which can be expected due to the much larger size of the nanoparticles (350 × 170 × 40 nm) compared to the case of smaller nanoparticles presented in Figure S5. This increased multipolar content is responsible for stronger hybridization of the LSPR with the TM-polarized guided-mode resonance, giving rise to two different quasi-BICs that can be efficiently excited at a slightly oblique incidence angle. Surface roughness (see Figure 2b) can further enhance the coupling of these modes to free-space radiation and reduce their *Q* factors.

## IAC Analysis

### Theoretical Model for IAC Lineshapes

To reproduce the single- and multiresonant features observed in the IAC curves in Figure 4, we develop a simple theoretical model^26,27,31,79−81,85,86,121^ that is consistent with the concept of quasi-normal modes^118^ in resonant optical systems. In this model, the local optical field *E*(*t*) is governed by the convolution of the incident field *E*_inc_(*t*) and the impulse response function *G*(*t*)
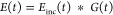
5

**Figure 6:**
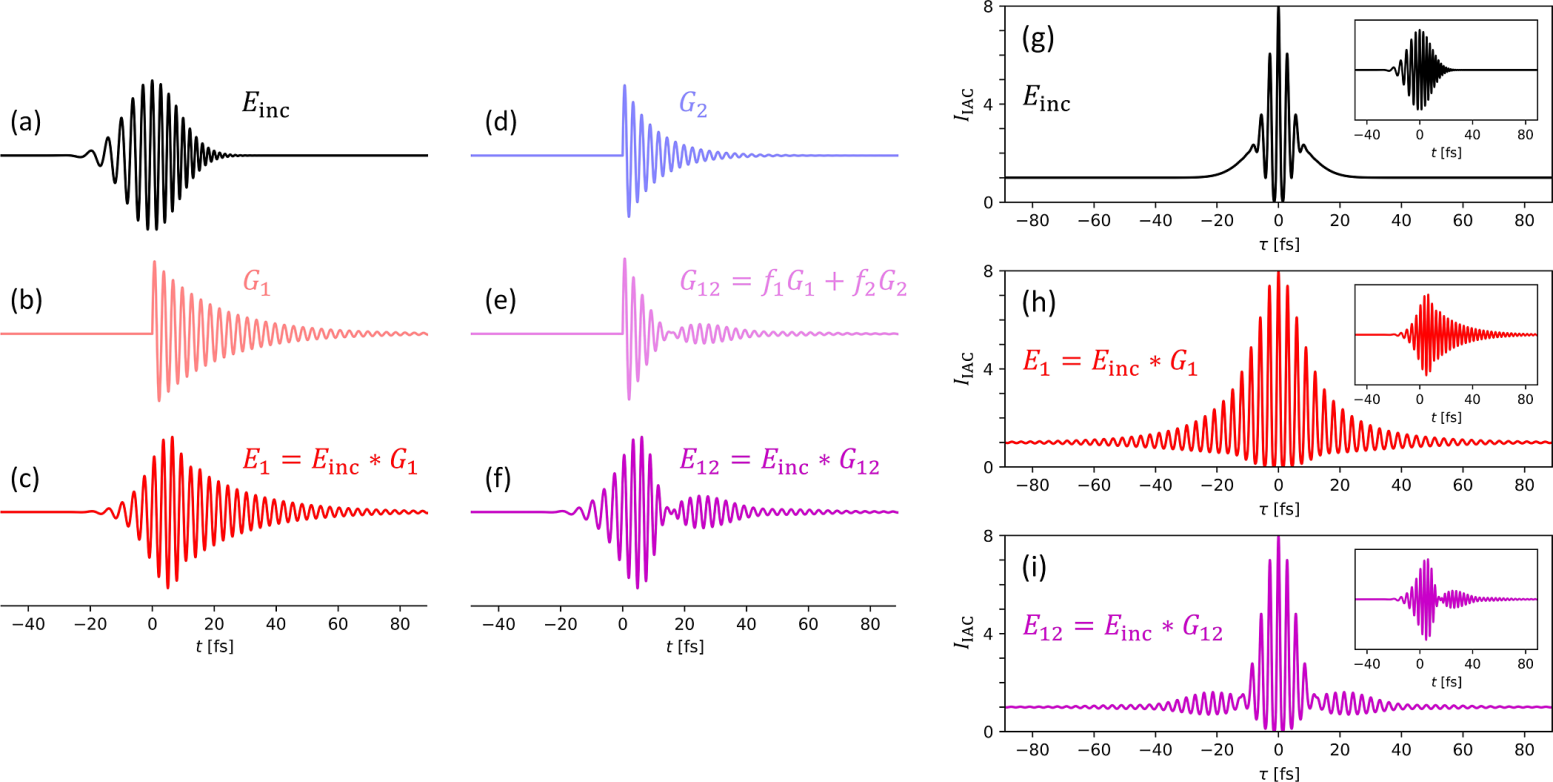
Examples illustrating the theoretical model: (a) incident field *E*_inc_(*t*), as described by eq 3 with ω = 2.2 rad/fs, *T* = 12 fs, and *C* = 0.01 rad/fs^2^; (b) impulse response function *G*_1_(*t*) due to a single resonance of Ω_1_ = 2.1 rad/fs and Γ_1_ = 0.05 rad/fs; (c) dynamics of the local optical fields due to resonance *G*_1_ driven by *E*_inc_(*t*); (d) impulse response function *G*_2_(*t*) due to a resonance of Ω_2_ = 2.3 rad/fs and Γ_2_ = 0.1 rad/fs; (e) impulse response function due to two resonances *G*_12_(*t*) = *f*_1_*G*_1_(*t*) + *f*_2_*G*_2_(*t*) with *f*_1_/*f*_2_ = 0.5; (f) dynamics due to the two resonances *G*_12_ driven by *E*_inc_(*t*); (g–i) theoretical IAC curves based on eq 1, with the local field *E*(*t*) governed by (g) *E*_inc_(*t*), (h) *E*_1_(*t*) = *E*_inc_(*t*)∗*G*_1_(*t*), and (i) *E*_12_(*t*) = *E*_inc_(*t*)∗*G*_12_(*t*). The insets in (g–i) show the corresponding plots of *E*(*t*), identical to those in (a,c,f), respectively.

To illustrate our model, we assume *E*_inc_(*t*) to take the form of a Gaussian pulse, as in eq 3, with the central frequency ω = 2.2 rad/fs (λ ≈ 857 nm), pulse duration *T* = 12 fs, and chirp *C* = 0.01 rad/fs^2^. Such exaggerated values of *T* and *C* are chosen to clearly reveal the main features of the temporal profile of the pulse, as can be seen in Figure 6a.

The impulse response function *G*(*t*) of a single resonance can be expressed as^26,121^
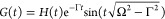
6where *H*(*t*) is the Heaviside step function, Ω is the resonance frequency, and Γ is the amplitude damping rate (Γ < Ω). The *Q* factor of such a resonance is *Q* = Ω/2Γ. Since the resonant response is considered in the time domain, all quantities in eqs 5 and 6 are real. Figure 6b shows an example of *G*(*t*), labeled as *G*_1_(*t*), corresponding to a resonance at Ω_1_ = 2.1 rad/fs (λ_1_ ≈ 900 nm) and Γ_1_ = 0.05 rad/fs (*Q* = 21). The temporal dynamics due to *G*_1_(*t*) driven by *E*_inc_(*t*) are shown in Figure 6c.

In general, optical systems may contain many resonances
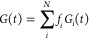
7each having its own resonance frequency Ω_*i*_ and damping rate Γ_*i*_, with the relative contributions determined by factors *f*_*i*_. As a simple example, we combine the previously considered *G*_1_(*t*) with a different resonance *G*_2_(*t*) (see Figure 6d) characterized by Ω_2_ = 2.3 rad/fs (λ_2_ ≈ 820 nm) and Γ_2_ = 0.1 rad/fs (*Q* = 11.5). Figure 6e shows *G*_12_(*t*) composed of the two resonances with *f*_1_/*f*_2_ = 0.5. The interference between *G*_1_(*t*) and *G*_2_(*t*) results in a beat pattern, which is visible in the response to the incident field in Figure 6f.

The capability of the IAC technique to reveal the resonant local field oscillations is illustrated in Figure 6g–i, in which the IAC curves are calculated using eq 1 assuming *E*(*t*) equal to *E*_inc_(*t*), *E*_1_(*t*), and *E*_12_(*t*), as in Figure 6a,c,f, respectively. Compared to the IAC curve for the incident field [*E*(*t*) = *E*_inc_(*t*), Figure 6g], the IAC curve for the resonantly enhanced field [*E*(*t*) = *E*_1_(*t*), Figure 6h] exhibits interference fringes at much larger delays τ. At resonance, the optical fields excited by the incident pulses get temporally stretched, which allows them to interfere with each other even if the incident pulses do not overlap in time. As opposed to the pulse chirping, in which the temporal stretching is caused by the delay between different frequency components, here the oscillations are temporally extended only at a given frequency. Furthermore, in the case of two resonances [*E*(*t*) = *E*_12_(*t*), Figure 6i], the IAC curve reveals the beat pattern that emerges due to interference between two different resonant frequencies (as in Figure 6e,f).

It is noteworthy that the incoherent character of TPEL greatly simplifies the use of the proposed theoretical model in the experimental IAC analysis. The lack of coherence makes the IAC signal insensitive to the interference effects related to the radiation pattern, polarization, or phase, which typically must be considered in the case of a coherent emission such as SHG.

### Fitting Experimental IAC Curves

We use the theoretical model introduced in the previous section to fit the experimental IAC curves that are clearly affected by the high-*Q* resonances. Since the parameters of the incident field are known from the reference measurements (Figure 3b,c), the remaining fitted parameters are the frequency (Ω_*i*_), damping rate (Γ_*i*_), and relative contribution (*f*_*i*_) of each resonance involved in the local field enhancement. Furthermore, we assume that the measured signal is a combination of the resonant and nonresonant contributions
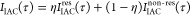
8where *I*_IAC_^res^(τ) corresponds to the enhanced TPEL from QDs inside the plasmonic hot spots of the nanoparticles, while *I*_IAC_^non-res^(τ) corresponds to the TPEL from QDs located in the “cold spots” in between the nanoparticles, where the emission is excited mainly by the incident fields (without the resonant enhancement). Both *I*_IAC_^res^(τ) and *I*_IAC_^non-res^(τ) are obtained using eq 1. However, *I*_IAC_^res^(τ) is based on the resonantly enhanced *E*(*t*), calculated using eqs 3 and 5–7, while *I*_IAC_^non-res^(τ) is identical to the theoretical reference IAC curve (see Figure 3b), calculated using eqs 1 and 3 with *T* = 150 fs and *C* = 5.5 × 10^–5^ rad/fs^2^. Thanks to the incoherent character of TPEL, both terms in eq 8 can be added together, and their relative contribution η becomes an additional fitted parameter.

**Figure 7:**
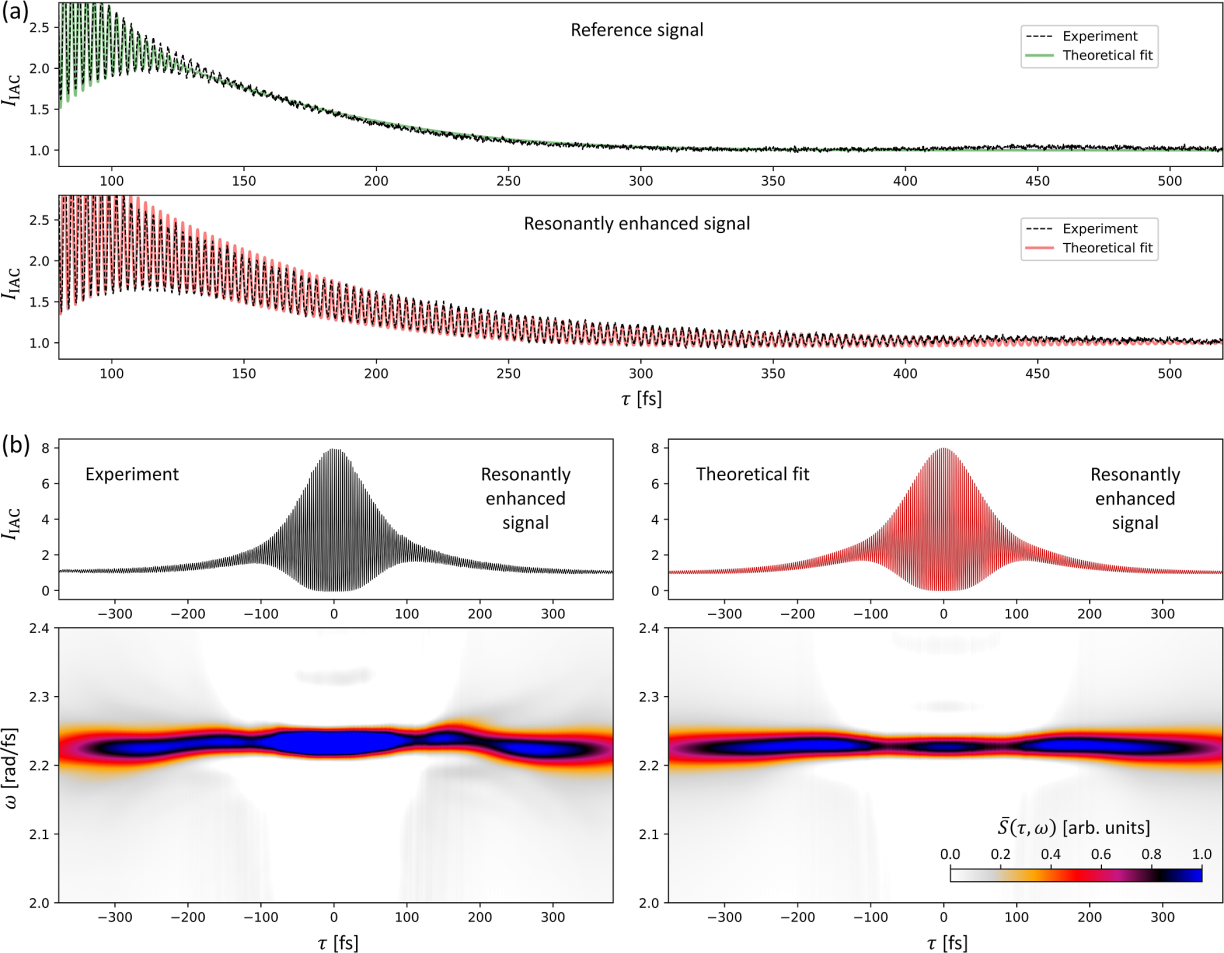
(a) Close-up views of the IAC curves, comparing the reference IAC curve (on the top, the same as in Figure 3b) and the IAC curve for the metasurface with *L* = 200 nm and Λ = 540 nm (on the bottom, part of the same IAC curve as in the second row in Figure 4a). The experimental curves are plotted in black, while the theoretical curves are either green (reference) or red (resonantly enhanced). Here, the reference IAC curve is vertically shifted by −0.15 to compensate for the flat background, which results from a longer exposure time necessary to measure the weaker signal from bare QDs. (b) Experimental (left/black) and theoretical (right/red) IAC curves (top row) and their spectrograms *S̅*(τ,ω) (bottom row) calculated using the procedure described in the “Methods” section, eqs 9–12, for the same metasurface as in (a). The theoretical IAC curve and the theoretical spectrogram were produced assuming the contribution of a single resonance with η = 0.2, Ω = 2.23 rad/fs (λ_res_ ≈ 844.7 nm), and Γ = 0.005 rad/fs (*Q* factor ≈ 223).

Figure 7a presents the analysis of the experimental IAC curve obtained for the metasurface with *L* = 200 nm and Λ = 540 nm (the same as in the second row in Figure 4a). In the reference IAC curve (Figure 7a, top, the same as in Figure 3b), the interference fringes disappear completely at τ > 250 fs. In contrast, in the IAC curve for the metasurface (Figure 7a, bottom), the fringes are visible even at τ as large as 500 fs. Since the amplitude of the fringes decays monotonically (without any beating), we can assume that the resonant response is dominated by a single high-*Q* resonance. This is in agreement with the numerical results presented in Figure 5a. The theoretical curve (red) is obtained assuming the contribution of a single resonance with the following parameters: η = 0.2, Ω = 2.23 rad/fs (λ_res_ ≈ 844.7 nm), and Γ = 0.005 rad/fs (*Q* factor ≈ 223). The relatively small contribution (20%) of the resonantly enhanced TPEL can be explained by the weak temporal overlap between the incident pulses and the impulse response function of the high-*Q* resonance, which hinders the coherent buildup of the local field amplitude,^26^ producing only a moderate resonant TPEL enhancement.

**Figure 8:**
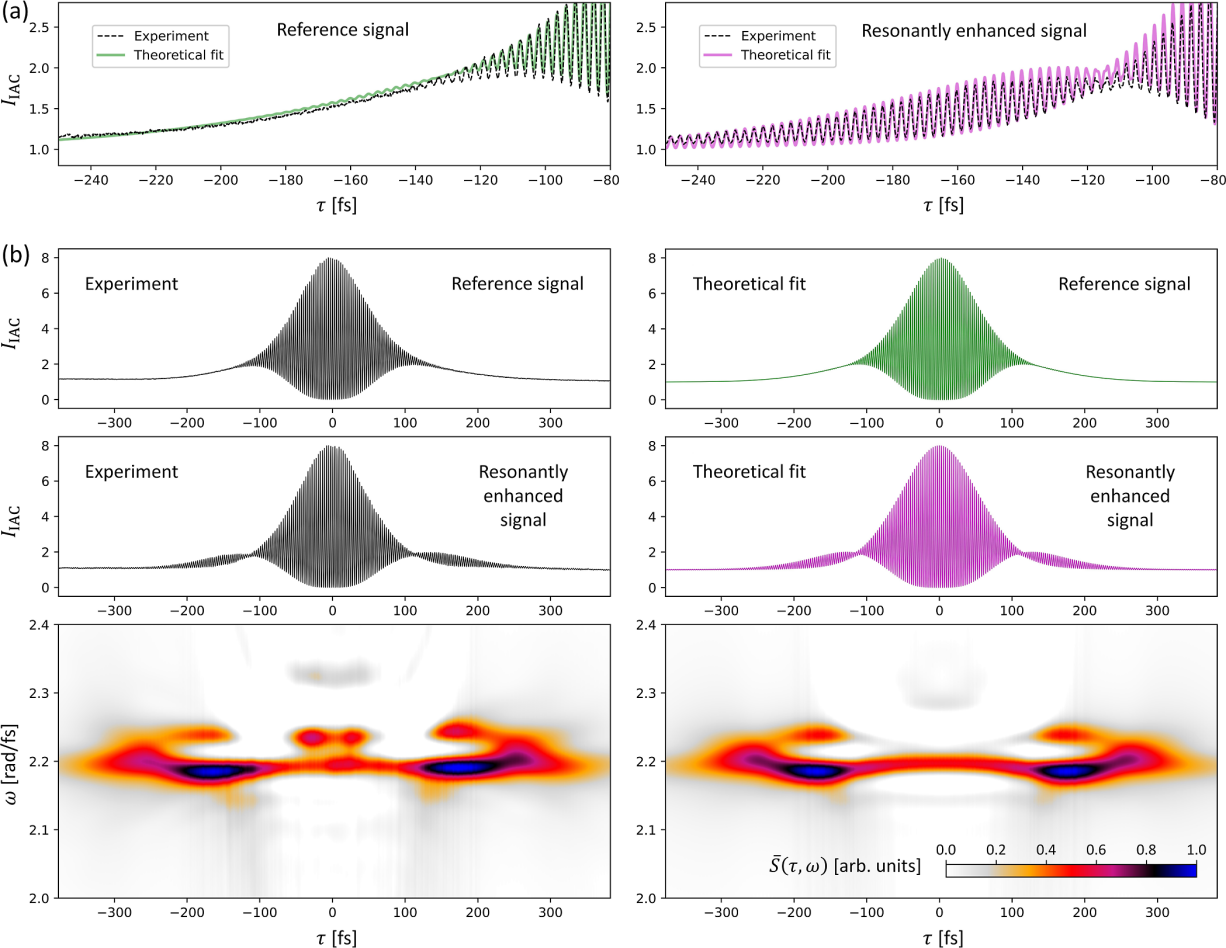
(a) Comparison between the reference IAC curve (left) and the IAC curve for the metasurface with *L* = 350 nm and Λ = 560 nm (right, part of the same IAC curve as in the fourth row in Figure 4b); black—experimental curves; green and magenta—theoretical curves. (b) Experimental and theoretical IAC curves for the reference (top row) and metasurface (middle row) and their corresponding spectrograms *S̅*(τ,ω) (bottom row). In this figure, the theoretical reference curve is the same as in Figures 3b and 7, but the experimental reference curve is different, as it was extracted from a different region of the TPEL images recorded during the reference IAC measurement (corresponding to the area of the illumination spot in which the metasurface was excited). Since the spectrogram is obtained by the deconvolution of the metasurface IAC spectrum vs the corresponding reference (see the “Methods” section), this reference is shown here. The theoretical IAC curve and the theoretical spectrogram were produced assuming the contributions of two resonances with η = 0.6129, Ω_1_ = 2.2005 rad/fs (λ_res_ ≈ 856.0 nm), Γ_1_ = 0.00985 rad/fs (*Q* factor ≈ 112), Ω_2_ = 2.2291 rad/fs (λ_res_ ≈ 845.0 nm), Γ_2_ = 0.019107 rad/fs (*Q* factor ≈ 58), and *f*_1_/*f*_2_ = 0.4477.

This fit-based analysis of IAC curves also provides quantitative insight in the multiresonant metasurfaces that show beat patterns in the IAC. In Figure 8a, we investigate the IAC curve obtained for the metasurface with *L* = 350 nm and Λ = 560 nm, which shows a very pronounced beat pattern (see Figure 4b, fourth row). The experimental IAC curve (black curve in Figure 8a) can be well reproduced by the theoretical curve (magenta) based on the simple model introduced in the previous section, assuming two resonances with η = 0.6129, Ω_1_ = 2.2005 rad/fs (λ_res_ ≈ 856.0 nm), Γ_1_ = 0.00985 rad/fs (*Q* factor ≈ 112), Ω_2_ = 2.2291 rad/fs (λ_res_ ≈ 845.0 nm), Γ_2_ = 0.019107 rad/fs (*Q* factor ≈ 58), and *f*_1_/*f*_2_ = 0.4477. The contribution of two resonances and the relative magnitudes of their *Q* factors agree qualitatively with the numerical results presented in Figure 5b.

We have found that the analysis of the IAC curves affected by the high-*Q* resonances can be greatly facilitated by converting them into spectrograms (method based on Fourier transform described in detail in the Methods section). Figures 7b and 8b (bottom row) show such spectrograms *S̅*(τ,ω) based on the experimental (left) and theoretical (right) IAC curves (top row) for the metasurface with a single resonance, as well as for the case with a strong resonance beating. In these diagrams, the time–delay axis (τ ≡ τ_0_) is horizontal, while the frequency axis (ω) is vertical. For the metasurface with just a single resonance (Figure 7b), the spectrograms, both in experiment and in theory, show that the resonant response of the metasurface is dominated by a single resonance at around 2.23 rad/fs. Figure 8b shows that for the metasurface with clearly beating resonances, the spectrogram clearly reveals distinctly separated spectral components. The experimental spectrogram is very well reproduced in our simple model.

We note that the Supporting Information contains a detailed analysis as a function of the metasurface lattice constant and nanoparticle size (Figures S7–S14), including all the corresponding reference IAC curves. These data rule out that the resonance-induced fringes and beat patterns are attributable to the optical elements in the setup and show that they are specific to the excitation conditions for (multiple) high-*Q* resonances. Additional examples of the fits (Figures S15–S20) demonstrate the impressive capability of the proposed IAC spectrograms to enhance the visibility of resonances hidden in the IAC curves.

## Conclusions

In this work, we used IAC imaging to reveal the temporal dynamics of optical fields enhanced by high-*Q* collective resonances in periodic metasurfaces. The studied resonances can be classified as both WPPs and multipolar quasi-BICs. An important advance is the use of CdSe/CdS/ZnS core/shell/shell QDs deposited on the nanoparticle arrays as nonlinear nanoprobes of the local optical fields, providing bright and stable IAC signals in the form of resonantly enhanced TPEL. The incoherent nature of TPEL is highly beneficial, as it allows to probe the resonantly enhanced near-fields at the fundamental frequency while avoiding the complications typically occurring at the nonlinear emission frequency for coherently emitted signals, such as the interference effects associated with their radiation patterns, polarizations, or phases.

The measured IAC curves revealed clear signatures of coherent oscillations in metasurfaces excited at the collective resonances, as well as the temporal beating due to the simultaneous excitation of two high-*Q* resonances at slightly different frequencies. Based on the measured IAC curves, we were able to determine the frequencies, the *Q* factors, and the relative contributions of the resonances to the TPEL enhancement. The experimentally determined *Q* factors were in the range from around 50 up to around 225. Finally, the unique interferometric stability of the fully common-path setup that uses a birefringent delay line for pulse separation is another key advantage of our approach. Combined with the excellent TPEL efficiency, it led to a very high quality of the measured IAC curves, allowing us to convert them into FROG-like spectrograms, which typically can only be obtained using coherent nonlinear effects such as SHG.

Our experimental and theoretical approaches can be generally applied to characterize the *Q* factors of diverse resonant optical systems, including resonant nano- and microstructures supporting high-Q photonic and plasmonic BICs. We expect that further developments of the proposed approaches, e.g., enabled by machine learning^75,122^ to categorize features in the information-rich IAC curves, could make them very powerful in studying resonant systems that exhibit complex temporal dynamics, such as anapole excitations, Rabi oscillations, Fano interference, and exotic effects of time-varying systems.

## Methods

### IAC Measurements

As the light source in our setup, we used a femtosecond laser (Light Conversion Twin-Orpheus F) with pulse duration *T* = 150 fs, repetition rate 1 MHz, and wavelength λ tunable in the range 780–920 nm. The wavelength was set to 850 nm during the IAC measurements.

To create pulse pairs with controllable time delay τ, we used the birefringent delay line^67^ shown in Figure 3a. The presented setup operates as follows. First, the polarization of the incoming pulses is set to 45° by a half-wave plate, such that upon transmission through a birefringent crystalline block (α-BBO) each pulse is split into two mutually delayed horizontally and vertically polarized pulses. Next, the two pulses travel through a pair of birefringent wedge prisms. The first prism causes polarization-dependent deflection, which is converted into transverse displacement by the second prism. In consequence, the two prisms together introduce a tiny difference between the optical paths of the two pulses. The pulses are then reflected back and follow the same paths through the birefringent elements, such that the delay introduced by these elements is doubled. By shifting one of the birefringent prisms in the transverse direction with a motorized translation stage, the overall delay between the pulses can be tuned from negative to positive (thanks to the delay offset provided by the birefringent block, the optic axis of which is oriented perpendicular to the optic axis of both prisms). The pulses exit the delay line through a nonpolarizing beam splitter and pass through a polarizer oriented at 45°, which converts them into the same polarization. The final polarization delivered to the sample is controlled by another half-wave plate.

The beam that illuminated the samples had an average power of 18.6 mW in the situation in which the two pulses perfectly overlapped in time (τ = 0). This value is reduced by a factor of 2 when the two pulses are strongly separated (τ ≫ *T*) due to the lack of coherent addition of their electric field amplitudes. The incident beam was slightly focused by a lens (*f* = 200 mm), such that the fwhm of the incident beam was around 180 μm in the sample plane (placed slightly out of the focal plane of the lens), yielding a peak intensity of 350 MW/cm^2^. The TPEL emitted by the sample was collected by an objective (NA = 0.7) after transmission through the transparent glass substrate. A set of short-pass filters rejected the fundamental beam, while the transmitted TPEL signal was imaged on a CCD camera, producing images such as those presented in the bottom row in Figure 2e. The IAC measurements of the metasurfaces were performed by collecting sequences of such TPEL images while scanning τ. Each of the images corresponds to a certain τ, and the whole sequence was used to retrieve a complete IAC curve for each metasurface, which was done by integrating the signal over the corresponding square regions of each image.

### TPEL Emission and Extinction Spectra

The TPEL emission spectra shown in Figure 2d were obtained by directing the signal from the objective to an optical fiber connected to a spectrometer. To measure the extinction spectra of the individual metasurfaces (see Figure 4, red curves, as well as Figures S4, S11 and S12 in the Supporting Information), the incident laser beam was replaced by a collimated beam of white light. In both configurations (for the TPEL emission and for the extinction measurements), the signal was spatially filtered by a pinhole positioned in the intermediate image plane (see Supporting Information, Figure S3), limiting the measured signal to that emitted from the areas corresponding to the selected metasurfaces. The presented values of the extinction were calculated with respect to the reference transmission through the sample areas away from the metasurfaces.

### TPEL Enhancement Spectra

Tuning the incident light wavelength allowed us to investigate the spectral dependence of the resonant TPEL enhancement. The dependence of TPEL enhancement on fundamental wavelength (Figure 4, blue curves, as well as Figures S4, S13 and S14 in the Supporting Information) was obtained by illuminating the sample with femtosecond pulses of different wavelengths in the range 780–920 nm and by wide-field imaging of the TPEL emission on the camera. The values of TPEL enhancement were calculated with respect to the reference values of the TPEL signal obtained from the areas without metasurfaces (from the layer of bare QDs under the SU-8 layer).

### IAC Spectrograms

In IAC curves, the fringes caused by resonant oscillations are periodic along τ, and their period is essentially equal to the duration of one oscillation cycle of the local field. Therefore, revealing the frequency components of the IAC curves can be helpful in estimating the number of contributing resonances and their frequencies. However, large contribution (1 – η) of the “nonresonant” signal (*I*_IAC_^non-res^, see eq 8) may obscure the desired information. There are two possible solutions to this problem: (1) resolving the IAC spectrum along τ, i.e., creating a spectrogram with τ along one of the axes, and (2) deconvolving the spectra of the metasurface-IAC versus the reference-IAC. Here, we propose a procedure that includes both solutions.

First, we select a single point τ_0_ on the IAC curve, and we define an interval of width Δτ around that point. Next, we calculate the spectrum *S*(τ_0_, Δτ, ω) of the IAC curve fragment within that interval
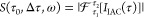
9where  is the discrete Fourier transform in the range from  to . Initially, the values of *S*(τ_0_, Δτ, ω) are obtained at frequencies ω determined by Δτ. To obtain the spectrum at frequencies ω′ independent of Δτ, we use cubic interpolation cub_ω′_(···)
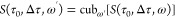
10

The above procedure is applied to both the metasurface-IAC and reference-IAC, yielding the corresponding spectra, *S*_M_(τ_0_, Δτ, ω′) and *S*_R_(τ_0_, Δτ, ω′), respectively. Next, we compute a deconvolution *S*_M/R_(τ_0_, Δτ, ω′) of the first versus the second spectrum using the following formula
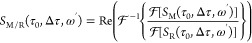
11where  and  are the discrete Fourier transform and discrete inverse Fourier transform, respectively, and the division is performed pointwise with complex-valued functions. Finally, we average *S*_M/R_(τ_0_, Δτ, ω′) over a large number (*N*) of the Δτ values spanning from Δτ_min_ to Δτ_max_ with a weighting factor 
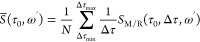
12

The final spectrum  can be calculated for the subsequent values of τ_0_, building a FROG-like spectrogram, with frequency ω′ on one axis and time delay τ_0_ on the other axis. The plots presented in Figures 7, 8, and S15–S20, were obtained by averaging over *N* = 1400 values of Δτ in the range from Δτ_min_ ≈ 11.16 fs (100 data points) to Δτ_max_ ≈ 167.45 fs (1500 data points). Negative values of *S̅*(τ,ω) are not shown in these plots due to saturation.

The patterns revealed in such spectrograms depend on various choices, such as the range of Δτ to average over. Nevertheless, we find this method helpful in the qualitative analysis of the resonances hidden in the IAC curves.

## Supporting Information Available

Spectra of the fundamental beam, SHG from a nonlinear crystal, and TPEL from QDs, as a function of the fundamental wavelength; power curves for the TPEL from QDs as a function of the fundamental wavelength, compared to the SHG at the fundamental wavelength of 820 nm; detailed scheme of the experimental setup; IAC curves, extinction spectra, and TPEL enhancement spectra, for the metasurfaces with *L* = 200 and 350 nm and Λ in the range 530–580 nm, for the incident beam polarized perpendicular to the long axes of the nanoparticles; electromagnetic multipole expansion for a nanoparticle inside the metasurface with *L* = 200 and 350 nm, Λ = 550 nm, calculated in COMSOL; IAC curves for the metasurfaces and the corresponding references, extinction spectra, and TPEL enhancement spectra, for all the metasurfaces under two different polarizations of the incident beam; and additional examples of the theoretical fits to the experimental IAC curves and their spectrograms (PDF)
