# Supplementary material for: Temporal Dynamics of Collective Resonances in Periodic Metasurfaces
Source: ACS Photonics. 2024 May 16;11(6):2480–96. doi: 10.1021/acsphotonics.4c00412 (PMC11191746; doi:10.1021/acsphotonics.4c00412)
Supplement: Supplementary file 1 — ph4c00412_si_001.pdf [file ph4c00412_si_001.pdf]

Supporting Information for: Temporal Dynamics of Collective Resonances in Periodic Metasurfaces

Radoslaw Kolkowski,[*,†,‡] Annemarie Berkhout,[‡] Sylvianne D. C. Roscam Abbing,[‡,¶] Debapriya Pal,[‡] Christian D. Dieleman,[§,¶] Jaco J. Geuchies,[‖] Arjan J. Houtepen,[‖] Bruno Ehrler,[§] and A. Femius Koenderink[*,‡,⊥]

†*Department of Applied Physics, Aalto University, P.O. Box 13500, Aalto FI-00076, Finland*

‡*Department of Physics of Information in Matter and Center for Nanophotonics, NWO-I Institute AMOLF, Science Park 104, Amsterdam, 1098 XG, The Netherlands*

¶*Advanced Research Center for Nanolithography (ARCNL), Science Park 106, Amsterdam, 1098 XG, The Netherlands*

§*Department of Sustainable Energy Materials and Center for Nanophotonics, NWO-I Institute AMOLF, Science Park 104, Amsterdam, 1098 XG, The Netherlands*

‖*Optoelectronic Materials Section, Faculty of Applied Sciences, Delft University of Technology, Van der Maasweg 9, Delft, 2629 HZ, The Netherlands*

⊥*Institute of Physics, University of Amsterdam, Amsterdam, 1098 XH, The Netherlands*

E-mail: radoslaw.kolkowski@aalto.fi; fkoenderink@amolf.nl

Contents

- **Figure S1** – Spectra of the fundamental beam, SHG from a nonlinear crystal, and TPEL from QDs, as a function of the fundamental wavelength.
- **Figure S2** – Power curves for the TPEL from QDs as a function of the fundamental wavelength, compared to the SHG at the fundamental wavelength of 820 nm.
- **Figure S3** – Detailed scheme of the experimental setup.
- **Figure S4** – IAC curves, extinction spectra, and TPEL enhancement spectra, for the metasurfaces with L = 200 and 350 nm and Λ in the range 530 - 580 nm, for the incident beam polarized perpendicular to the long axes of the nanoparticles.
- **Figures S5 and S6** – Electromagnetic multipole expansion for a nanoparticle inside the metasurface with L = 200 and 350 nm, Λ = 550 nm, calculated in COMSOL.
- **Figures S7 - S14** – IAC curves for the metasurfaces and the corresponding references, extinction spectra, and TPEL enhancement spectra, for all the metasurfaces under two different polarizations of the incident beam.
- **Figures S15 - S20** – Additional examples of the theoretical fits to the experimental IAC curves and their spectrograms.

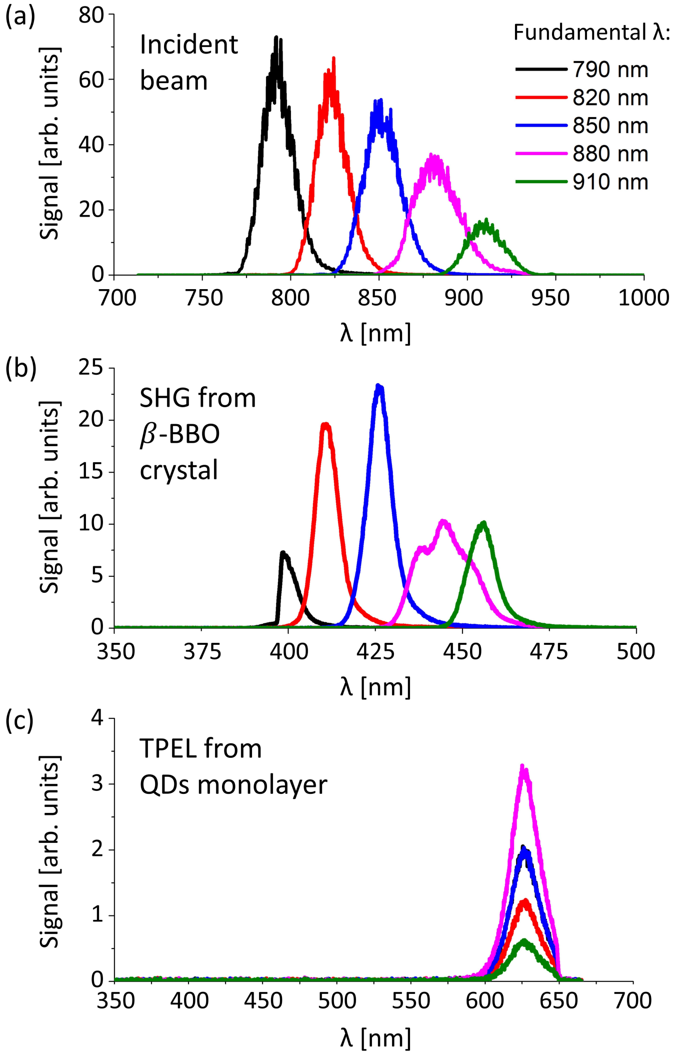

Figure S1: Signal spectra measured for different fundamental wavelengths in the range 790 - 910 nm, as indicated by the colors in the legend; (a) incident laser pulses; (b) SHG from a nonlinear crystal (β-BBO, thickness 10 μm); (c) TPEL from CdSe/CdS/ZnS core/shell/shell QDs (layer thickness 16 nm). Contrary to SHG, the line shape and spectral position of the TPEL emission peak do not depend on the fundamental wavelength. Furthermore, the spectra in (c) clearly show that TPEL is the only contribution to the QDs emission. In particular, the QDs do not emit any detectable SHG under the applied excitation conditions, since no peaks are visible in the range from 395 to 455 nm. Abrupt changes around 400 and 650 nm in some of the spectra are caused by the transmission edges of the filters used in the measurements.

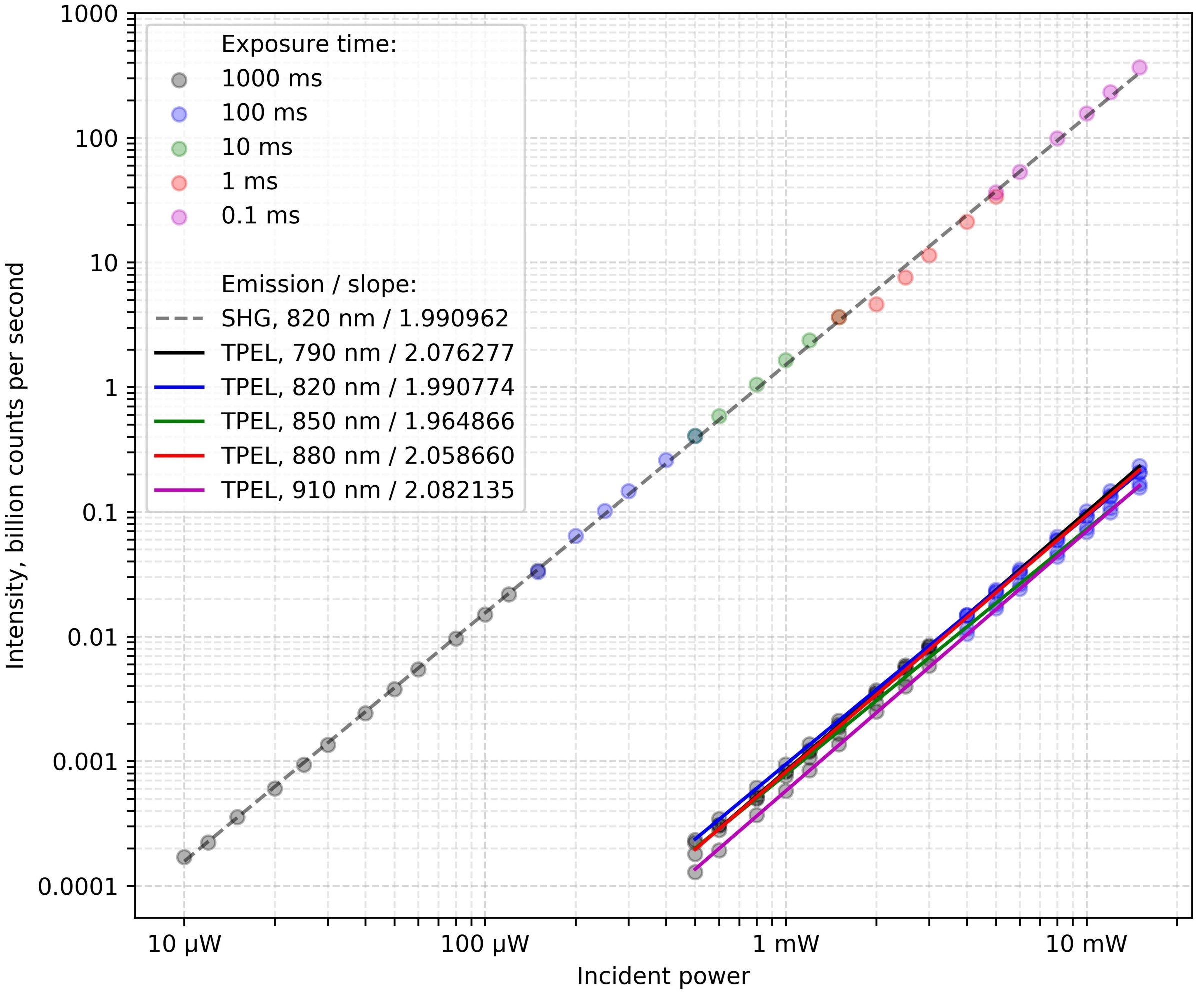

Figure S2: Power curves for the TPEL emitted by QDs, excited by the laser pulses of different fundamental wavelengths in the range 790 - 910 nm. The fundamental wavelengths are encoded in the colors of the fitting curves (solid lines). For comparison, we also show the power curve for the SHG emitted by a nonlinear crystal (excited at a fundamental wavelength of 820 nm), which is fitted by the dashed grey line. The color of the experimental data points (filled circles) encodes the exposure time. The slope of each curve in the log-log plot is shown in the legend. In all the cases, it is close to 2, which confirms the quadratic power dependence of the TPEL emitted by QDs.

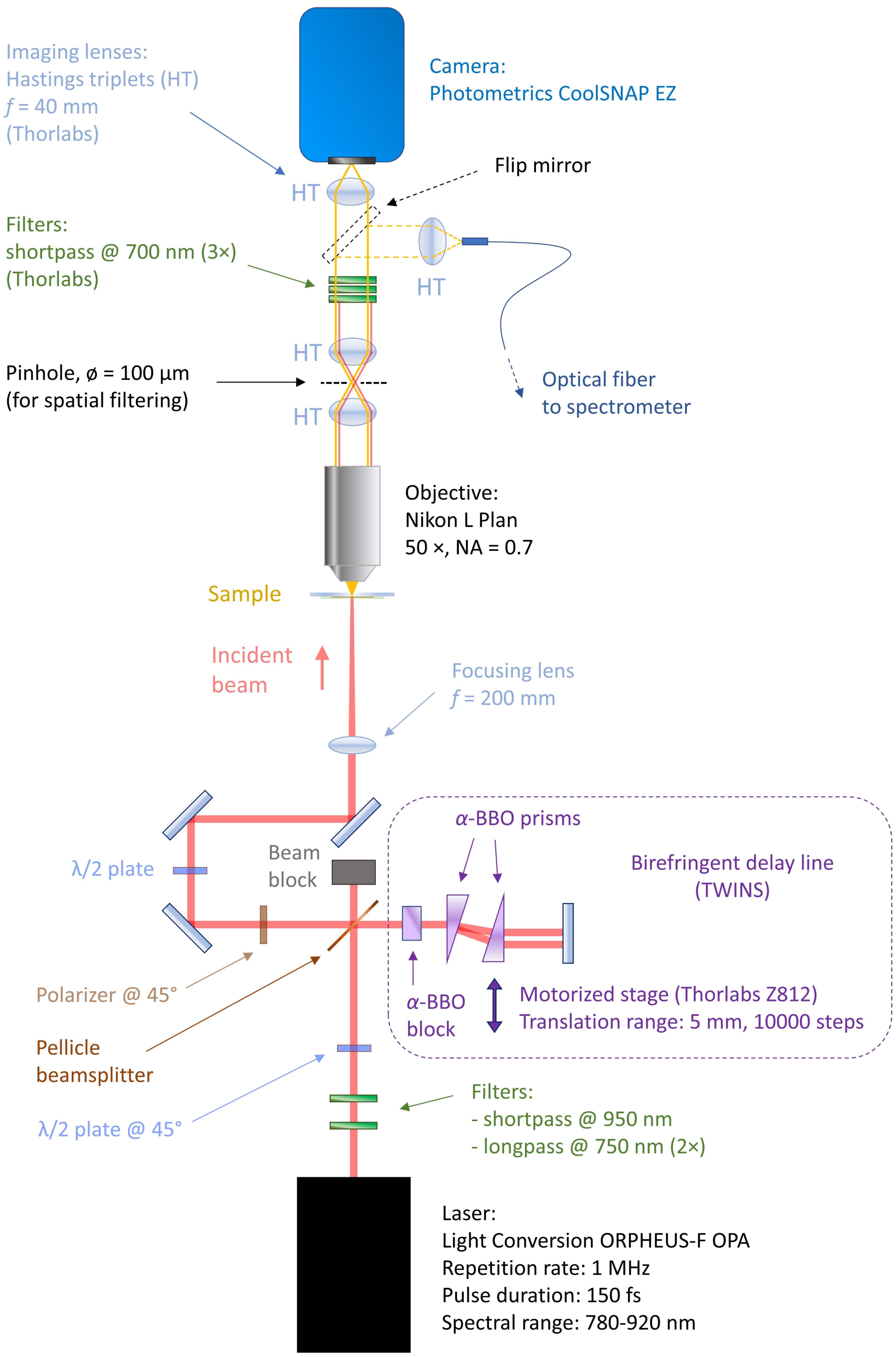

Figure S3: Detailed scheme of the experimental setup. The specified set of filters is used in the IAC measurements, but some of the filters are changed or removed in other types of measurements. The pinhole, located in the intermediate image plane, is used only during the measurements of the TPEL emission and white-light extinction spectra (Figure 2d and Figure 4 - red curves). In both of these measurements, the signal is directed by a flip mirror into an optical fiber connected to a spectrometer. In the extinction measurements, the incident femtosecond laser beam is replaced by a collimated beam from a broadband source (not shown in the scheme) bypassing the delay line.

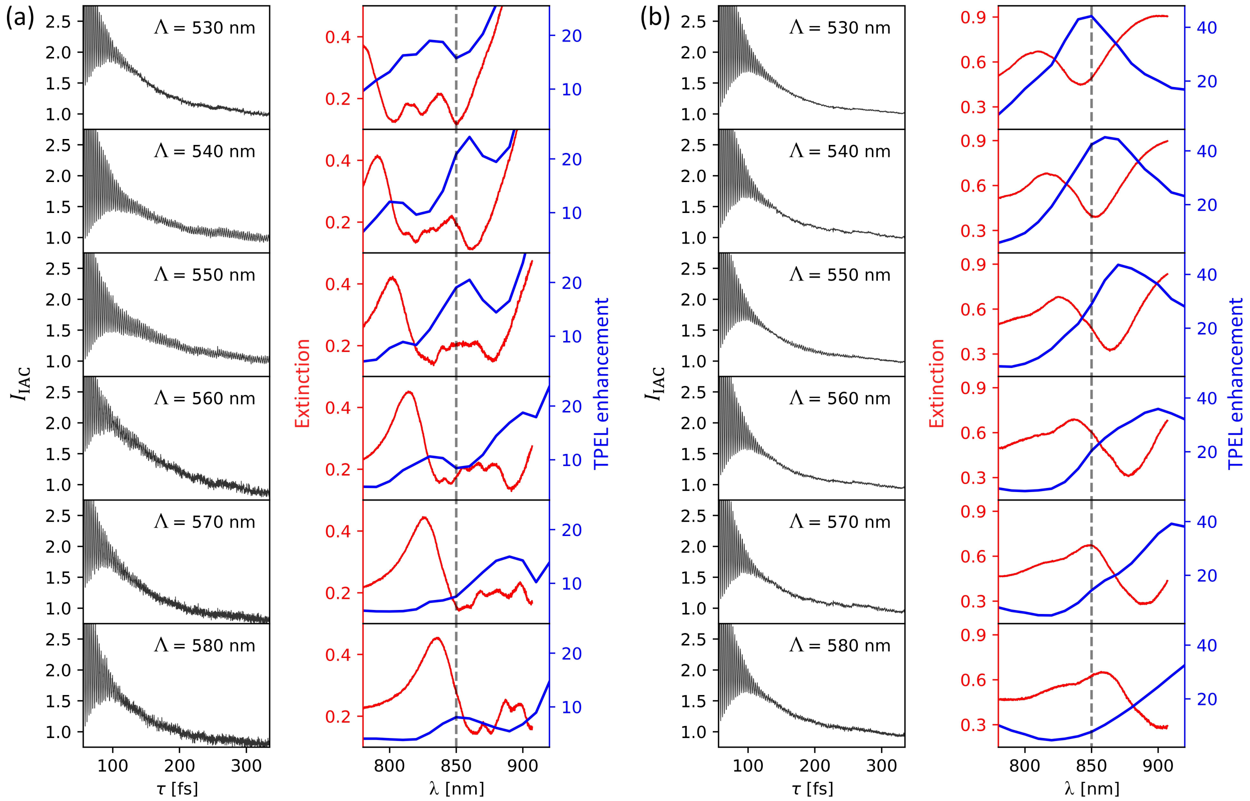

Figure S4: Results analogous to those presented in Figure 4, but obtained under the opposite polarization (perpendicular to the long axes of the nanoparticles, corresponding to the horizontal polarization in Figure 2e) for the nanoparticles of L = 200 (a) and L = 350 nm (b) as a function of Λ; left columns: close-ups of the measured IAC traces (black); right columns: extinction spectra (red) and TPEL enhancement spectra (blue). The dashed vertical line marks $\lambda = 850$ nm at which the IAC curves have been measured. Despite the presence of the resonant peaks in both the extinction and TPEL enhancement spectra, the IAC curves for L = 350 nm (b) do not show significant resonance-induced interference fringes, which is in sharp contrast to Figure 4.

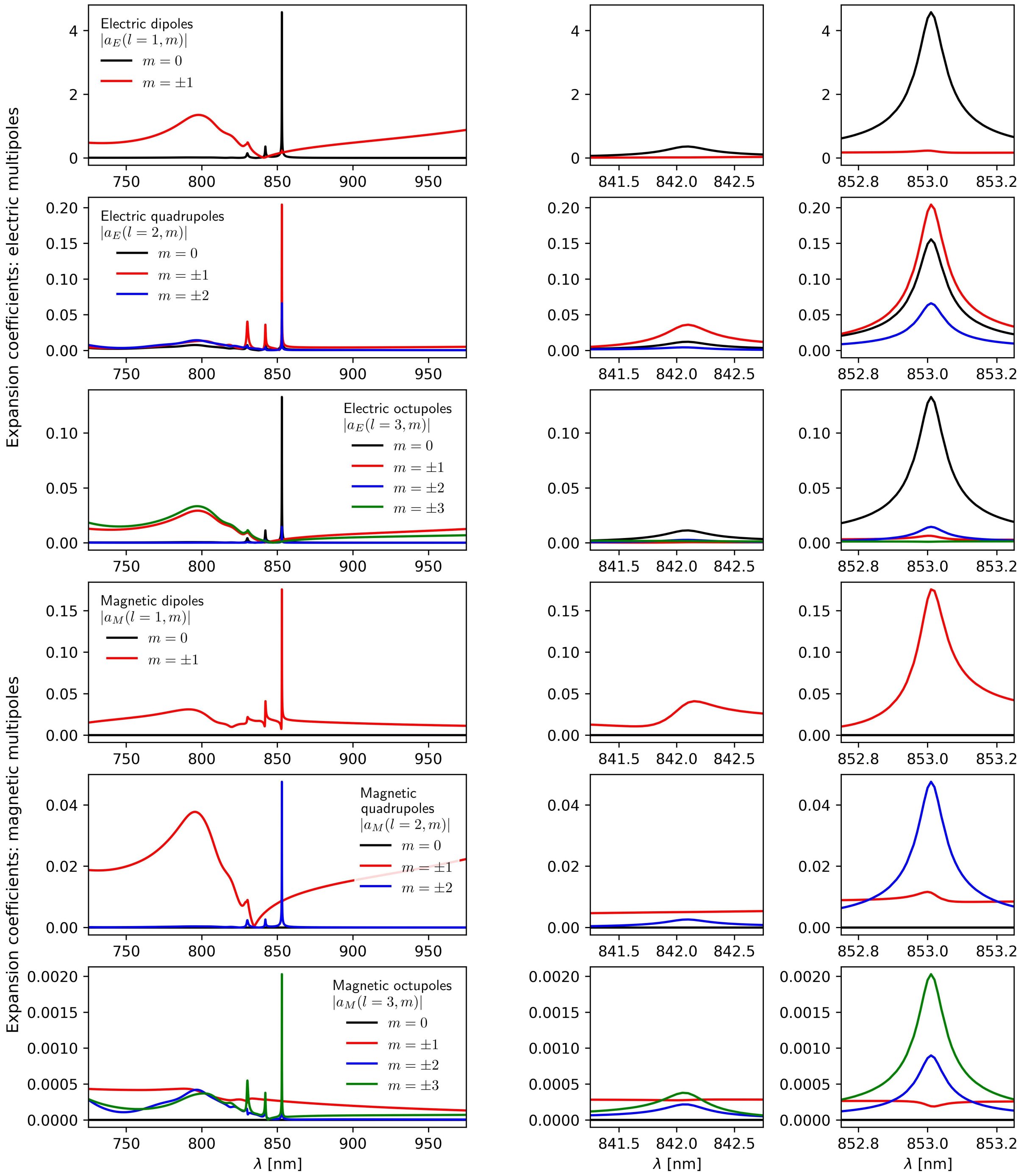

Figure S5: Electromagnetic multipole expansion for a single nanoparticle inside the metasurface with L = 200 nm, Λ = 550 nm (the same as in Figure 5a), illuminated by a plane wave propagating from top to bottom (mainly along the z-axis), linearly polarized in the xz-plane, mainly along the x-axis (parallel to the long axis of the nanoparticle), at incidence angle $\theta_x = 1^\circ$, $\theta_y = 0^\circ$. The spectral dependence is dominated by a single peak with strong out-of-plane electric dipole $a_E(l = 1, m = 0)$ due to hybridization with a TM-polarized guided-mode resonance.

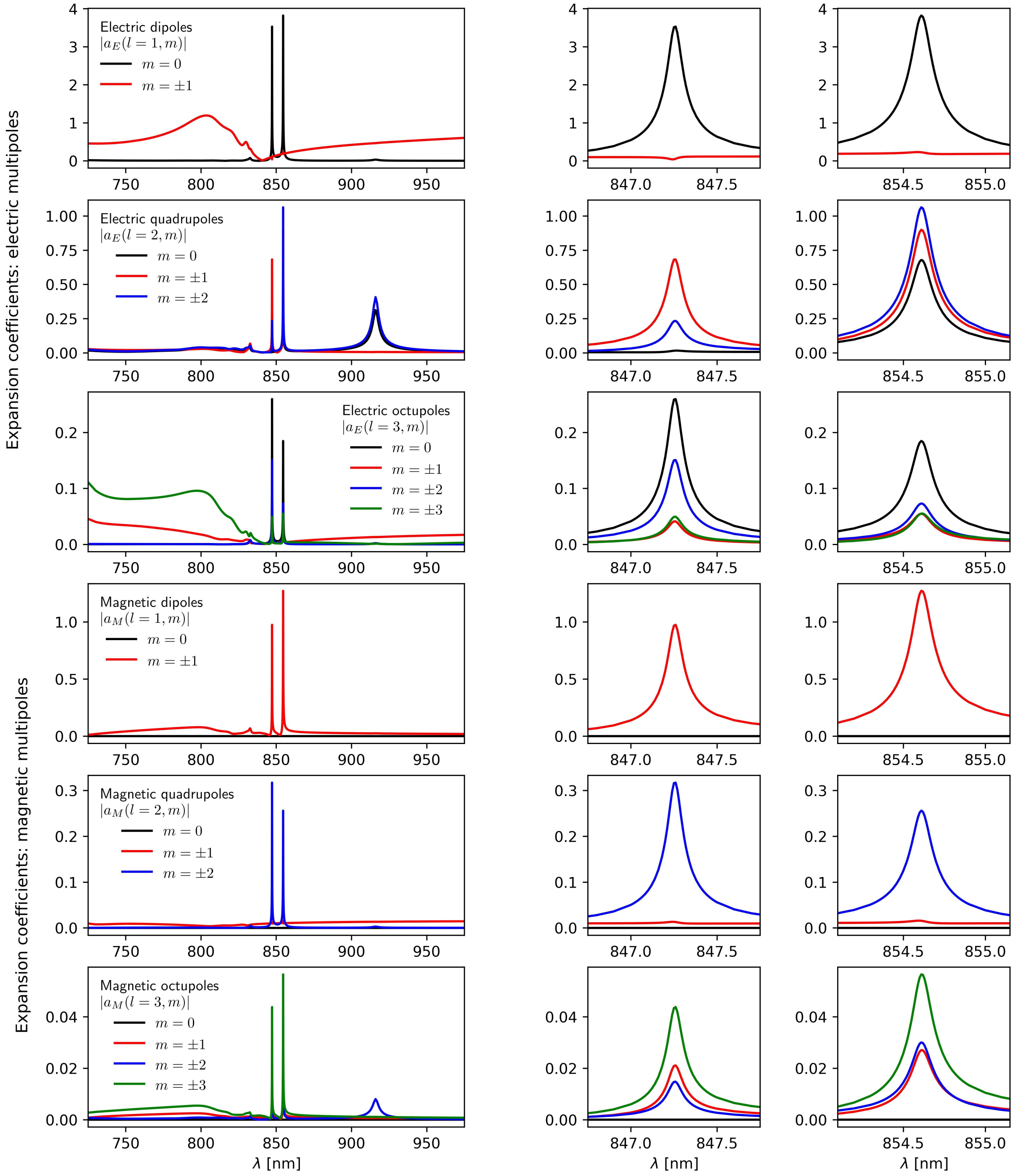

Figure S6: The same as in Figure S5, but for L $=350$ nm, corresponding to the case presented in Figure 5b with $\theta_x=1^\circ$, $\theta_y=0^\circ$. In contrast to Figure S5, the spectral dependence reveals two peaks of comparable magnitude and with significant contributions of electric quadrupoles and magnetic dipoles.

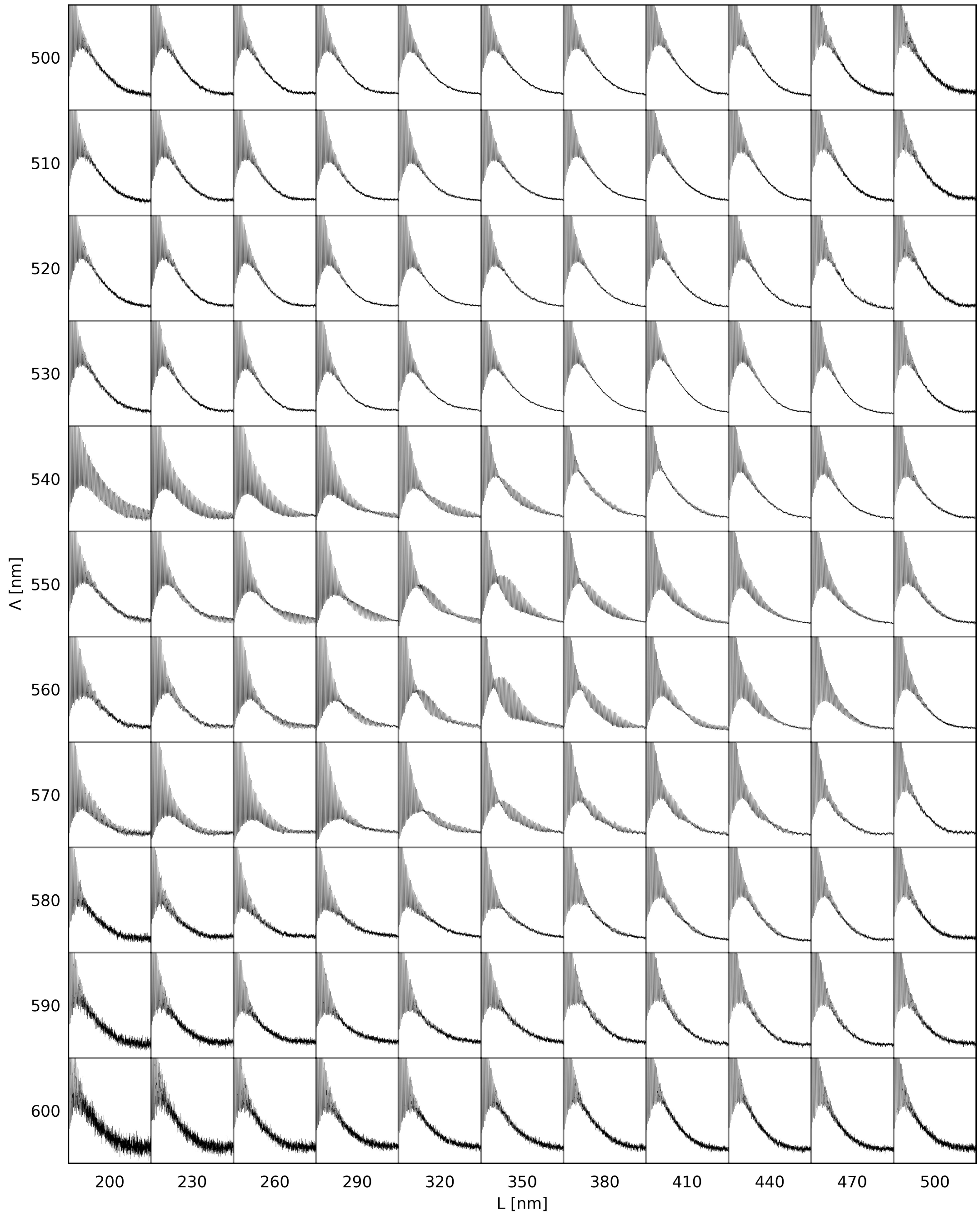

Figure S7: IAC curves measured for all the studied metasurfaces (nanoparticle lengths L ranging from 200 to 500 nm, lattice periods Λ from 500 to 600 nm), for the incident light polarization parallel to the long axis of the nanoparticles. The vertical axis of each plot spans from 0.75 to 2.75, while the horizontal axis represents the delay τ in the range from 70 to 350 fs (the same as in Figures 4 and S4).

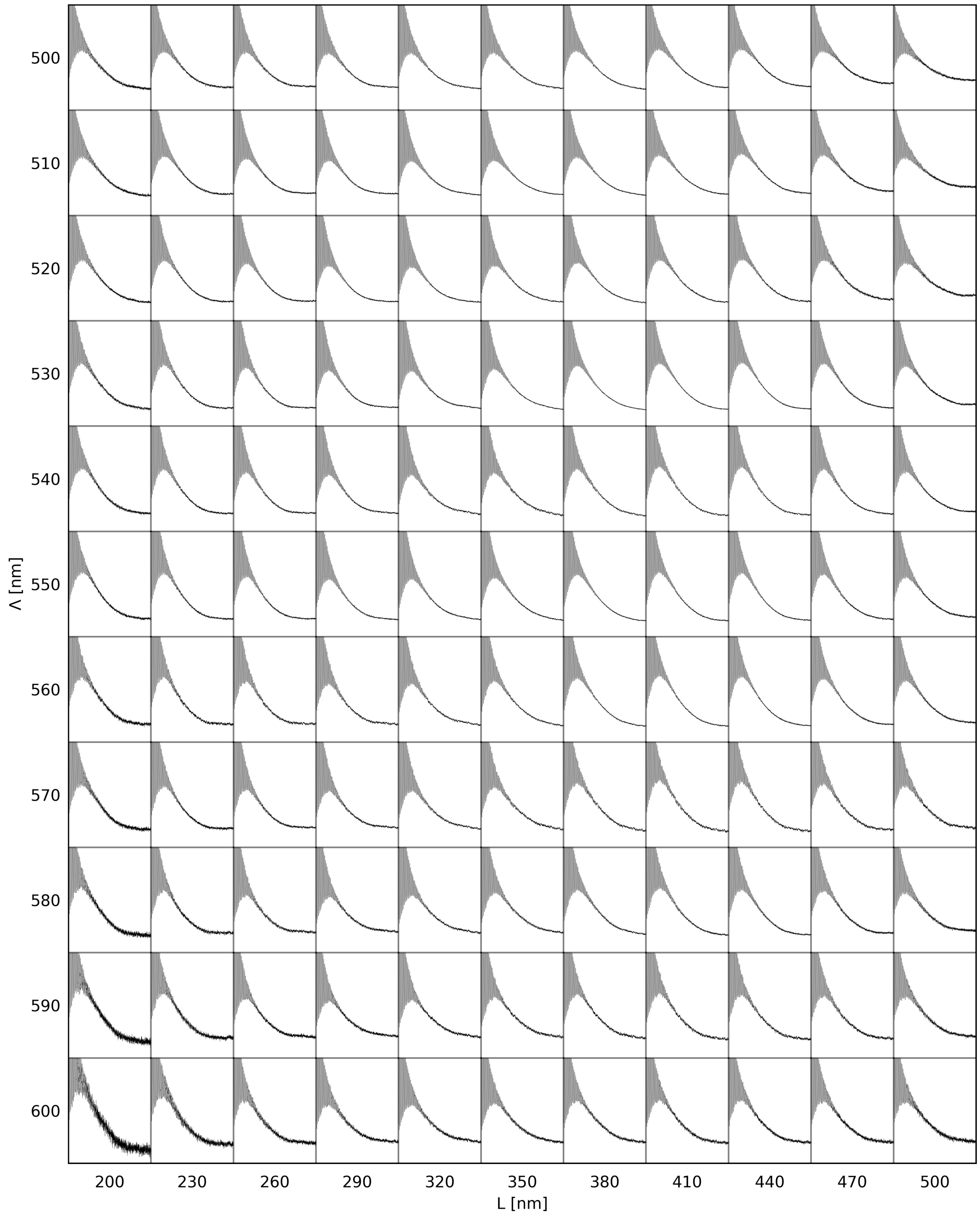

Figure S8: Reference-IAC curves measured on bare QDs without metasurfaces under the same incident light polarization as in Figure S7. For each of these curves, the TPEL signal was integrated over the same region of the camera images as for the corresponding metasurface-IAC curve in Figure S7.

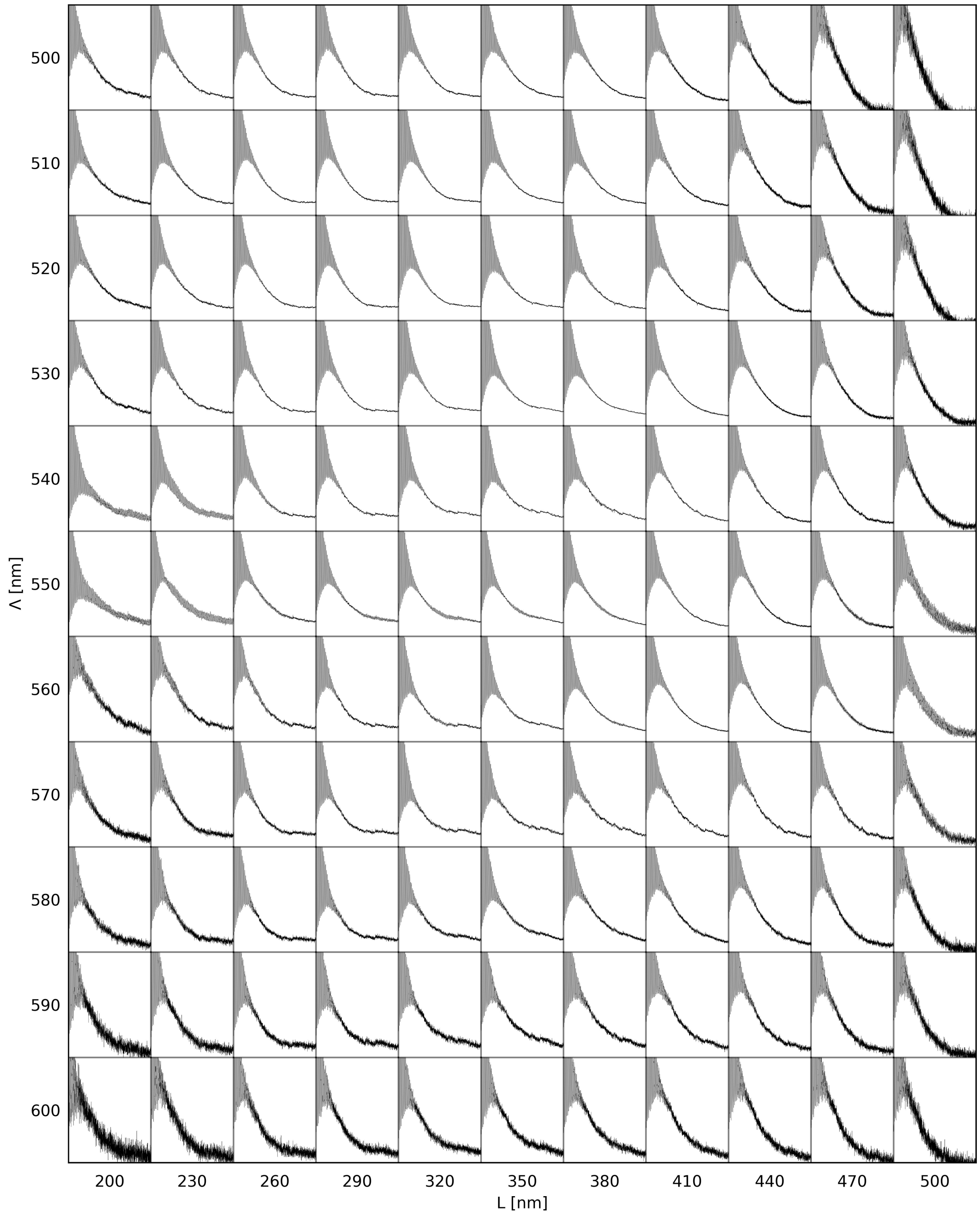

Figure S9: Metasurface-IAC curves measured under polarization perpendicular to the long axis of the nanoparticles (opposite to that in Figures S7 and S8). The layout of the plots is the same as in Figure S7.

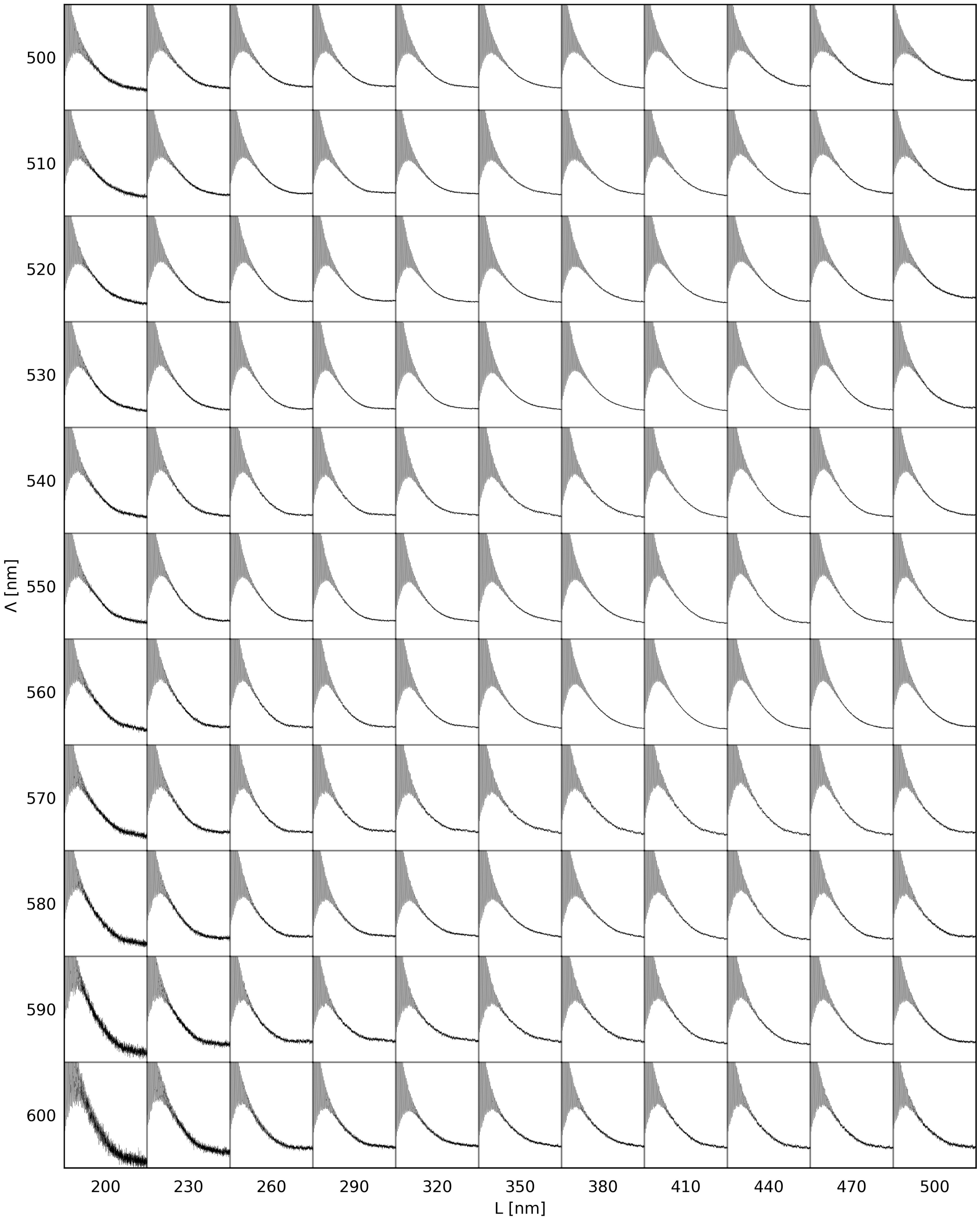

Figure S10: Reference-IAC curves for the metasurface-IAC curves in Figure S9 (analogous to Figures S7 and S8).

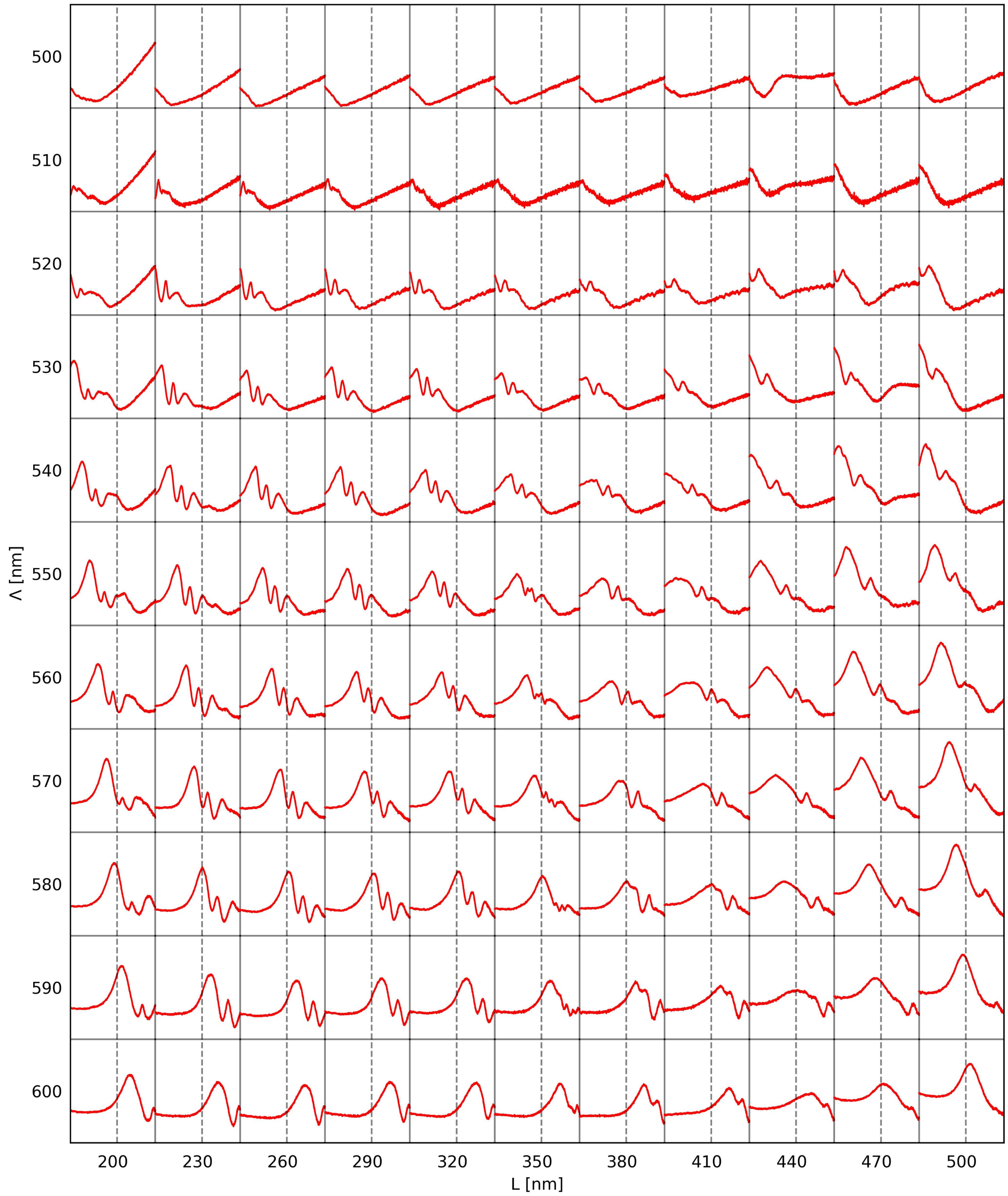

Figure S11: Extinction spectra in the wavelength range 780 - 920 nm (dashed line = 850 nm), for the incident light polarized parallel to the long axis of the nanoparticles. The vertical axis of each plot spans from 0 to 0.6.

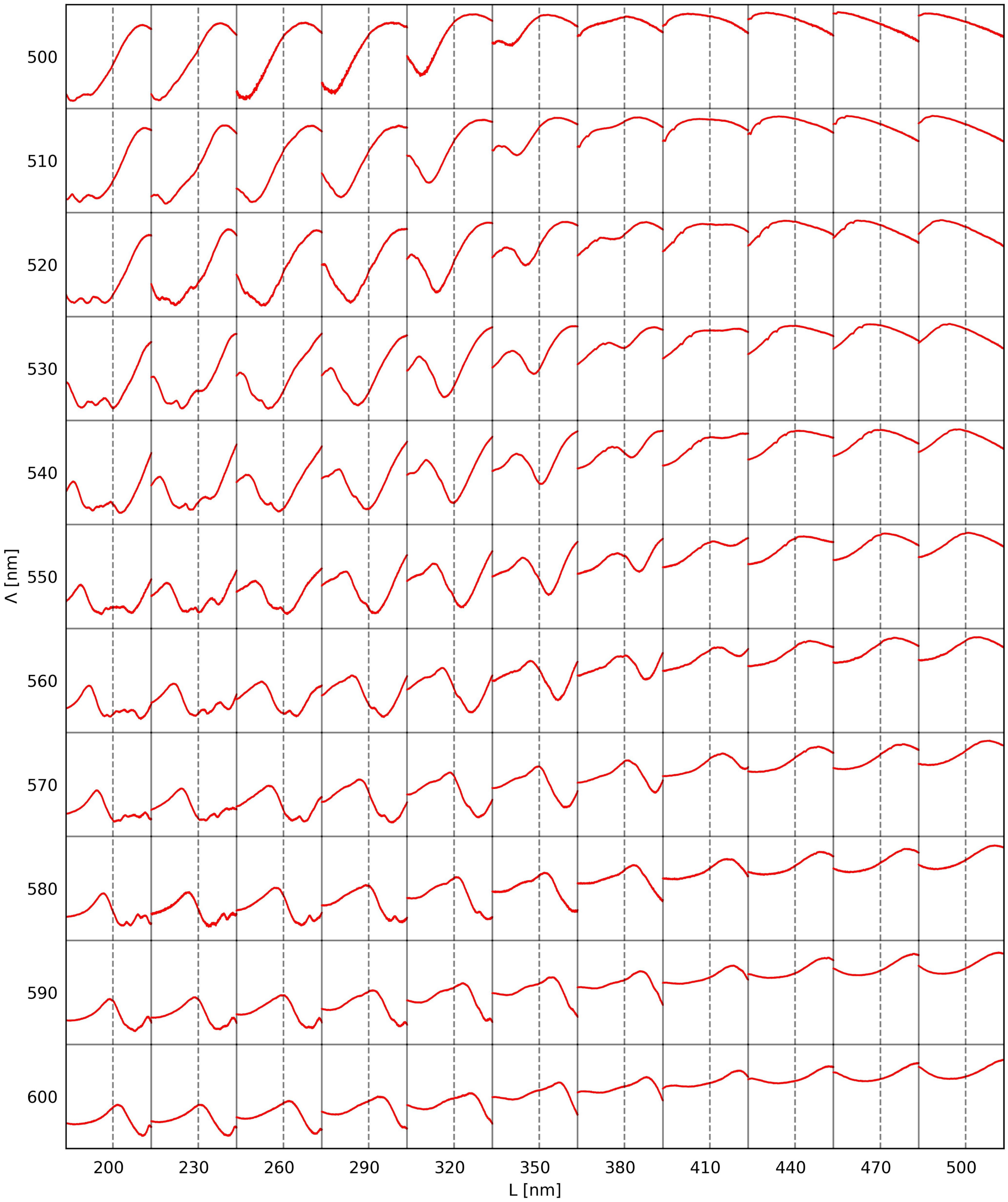

Figure S12: Extinction spectra in the wavelength range 780 - 920 nm (dashed line = 850 nm), for the incident light polarized perpendicular to the long axis of the nanoparticles. The vertical axis of each plot spans from 0 to 1.

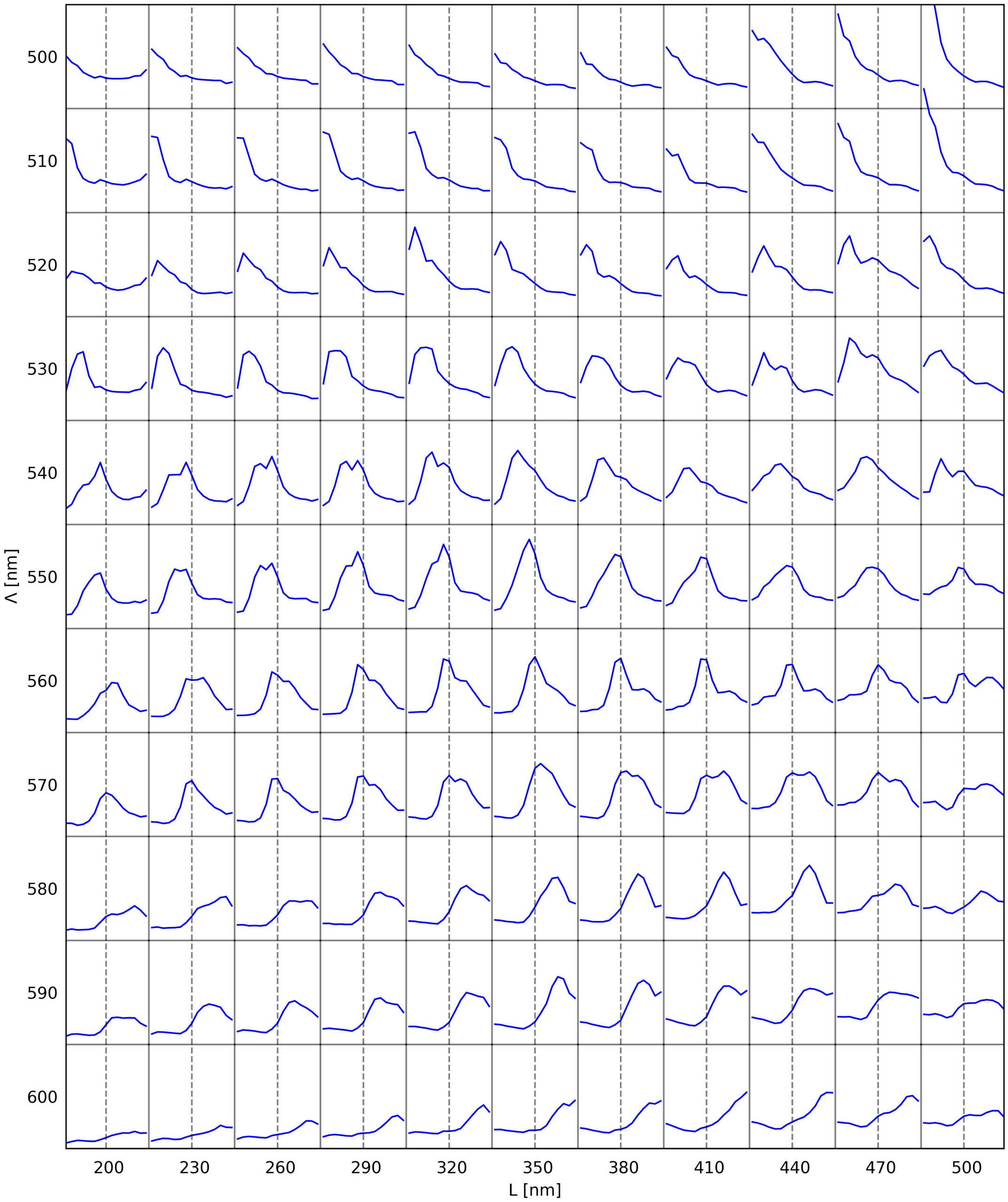

Figure S13: TPEL enhancement spectra in the wavelength range 780 - 920 nm (dashed line = 850 nm), for the incident light polarized parallel to the long axis of the nanoparticles. The vertical axis of each plot spans from 0 to 25.

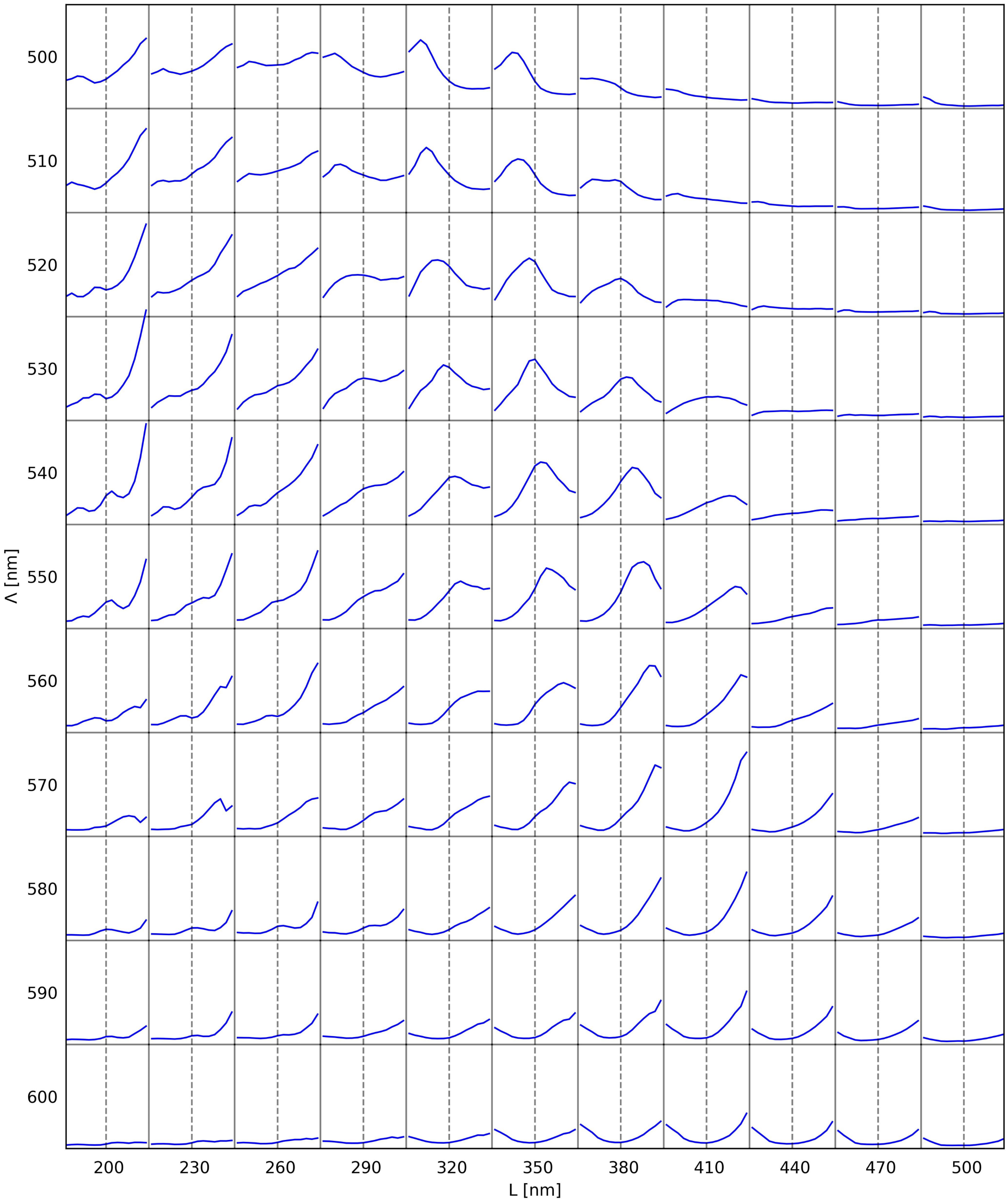

Figure S14: TPEL enhancement spectra in the wavelength range 780 - 920 nm (dashed line = 850 nm), for the incident light polarized perpendicular to the long axis of the nanoparticles. The vertical axis of each plot spans from 0 to 75.

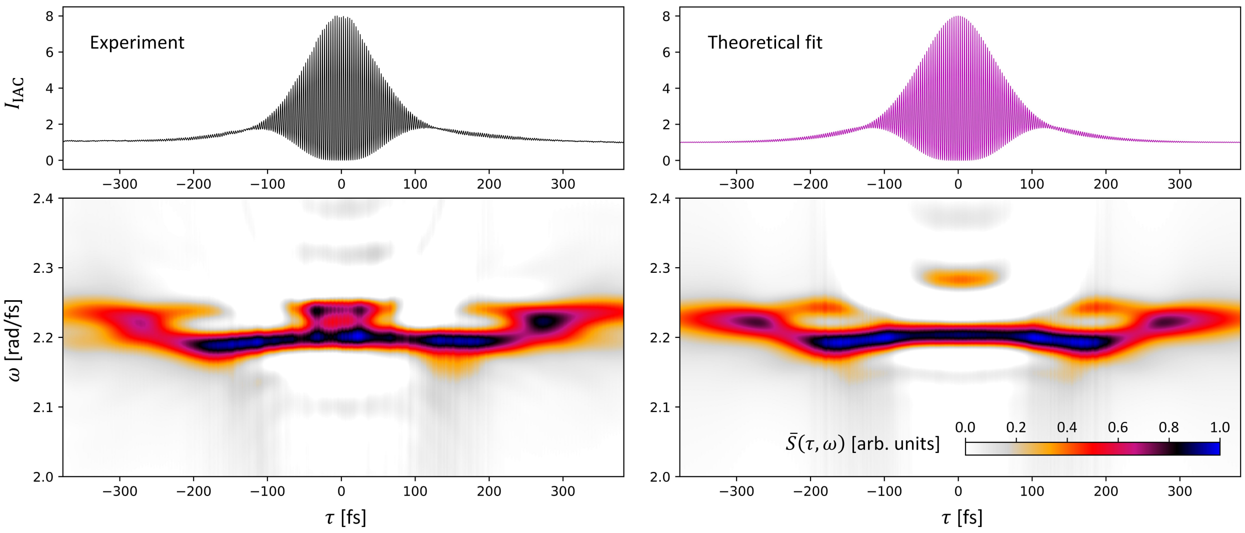

Figure S15: Experimental IAC curve (top left) and its spectrogram (bottom left), together with their theoretical fits (top right and bottom right, respectively), for the metasurface with L = 350 nm and Λ = 540 nm, under the incident light polarized parallel to the long axis of the nanoparticles. Fitted parameters: $\eta = 0.3369$, $\Omega_1 = 2.2075$ rad/fs ($\lambda_{res} \approx 853.3$ nm), $\Gamma_1 = 0.0177$ rad/fs (Q factor $\approx$ 62), $\Omega_2 = 2.2325$ rad/fs ($\lambda_{res} \approx 843.7$ nm), $\Gamma_2 = 0.008584$ rad/fs (Q factor $\approx$ 130), and $f_1/f_2 = 3.6179$.

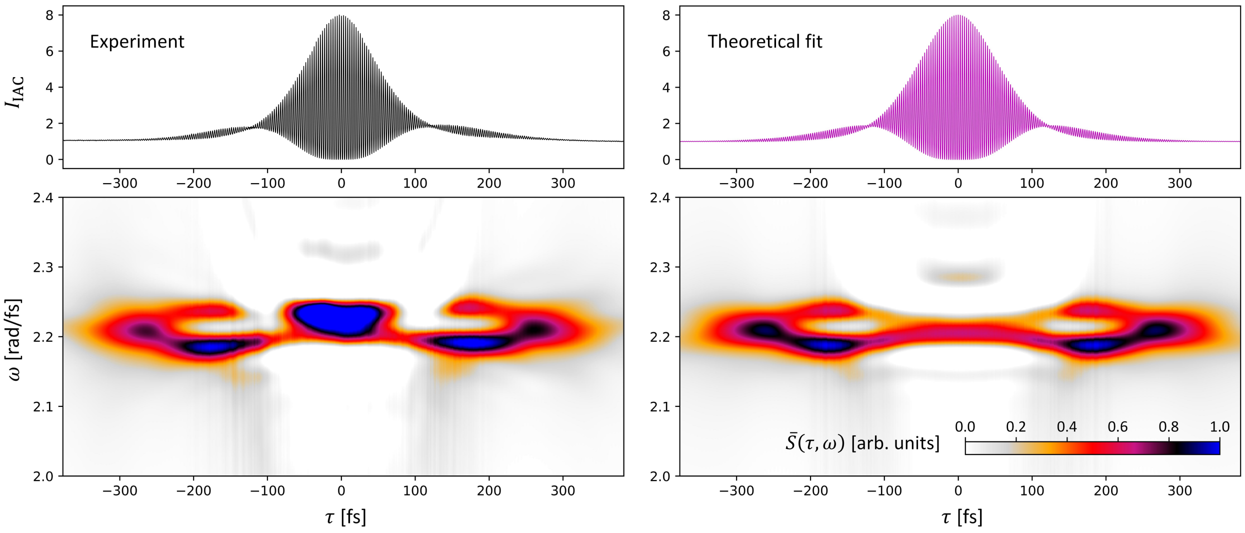

Figure S16: Experimental IAC curve (top left) and its spectrogram (bottom left), together with their theoretical fits (top right and bottom right, respectively), for the metasurface with L = 350 nm and Λ = 550 nm, under the incident light polarized parallel to the long axis of the nanoparticles. Fitted parameters: $\eta = 0.4857$, $\Omega_1 = 2.2035$ rad/fs ($\lambda_{res} \approx 854.8$ nm), Γ_1 = 0.00985 rad/fs (Q factor $\approx$ 112), $\Omega_2 = 2.2287$ rad/fs ($\lambda_{res} \approx 845.2$ nm), $\Gamma_2 = 0.014312$ rad/fs (Q factor $\approx$ 78), and $f_1/f_2 = 0.5973$.

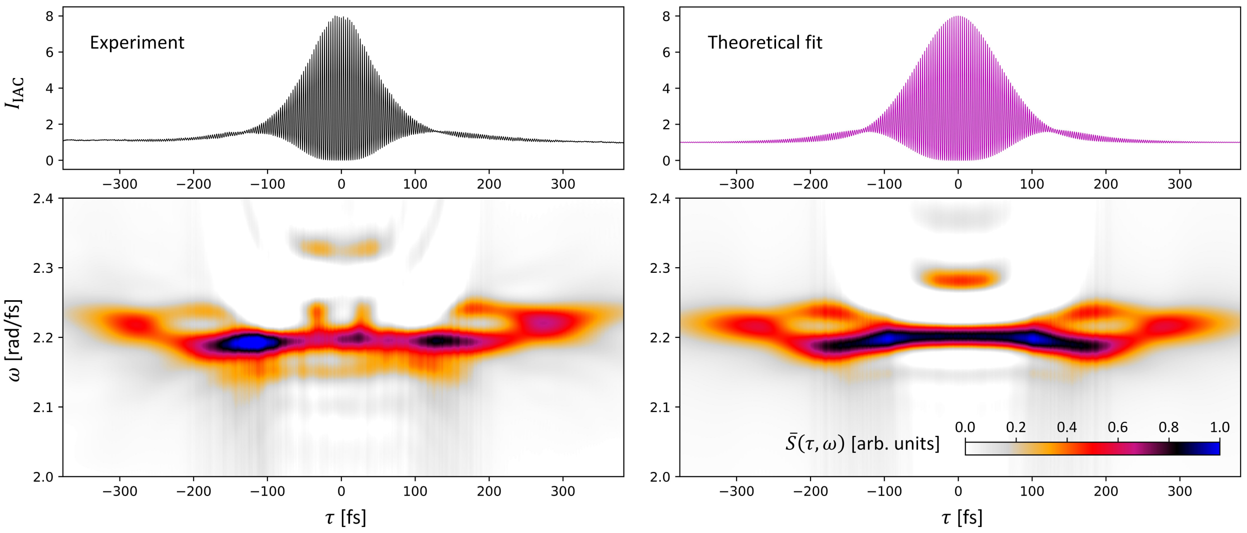

Figure S17: Experimental IAC curve (top left) and its spectrogram (bottom left), together with their theoretical fits (top right and bottom right, respectively), for the metasurface with L = 350 nm and Λ = 570 nm, under the incident light polarized parallel to the long axis of the nanoparticles. Fitted parameters: $\eta = 0.7479$, $\Omega_1 = 2.2275$ rad/fs ($\lambda_{\text{res}} \approx 845.6$ nm), $\Gamma_1 = 0.012$ rad/fs (Q factor ≈ 93), $\Omega_2 = 2.2034$ rad/fs ($\lambda_{\text{res}} \approx 854.9$ nm), $\Gamma_2 = 0.014312$ rad/fs (Q factor ≈ 61), and $f_1/f_2 = 0.4102$.

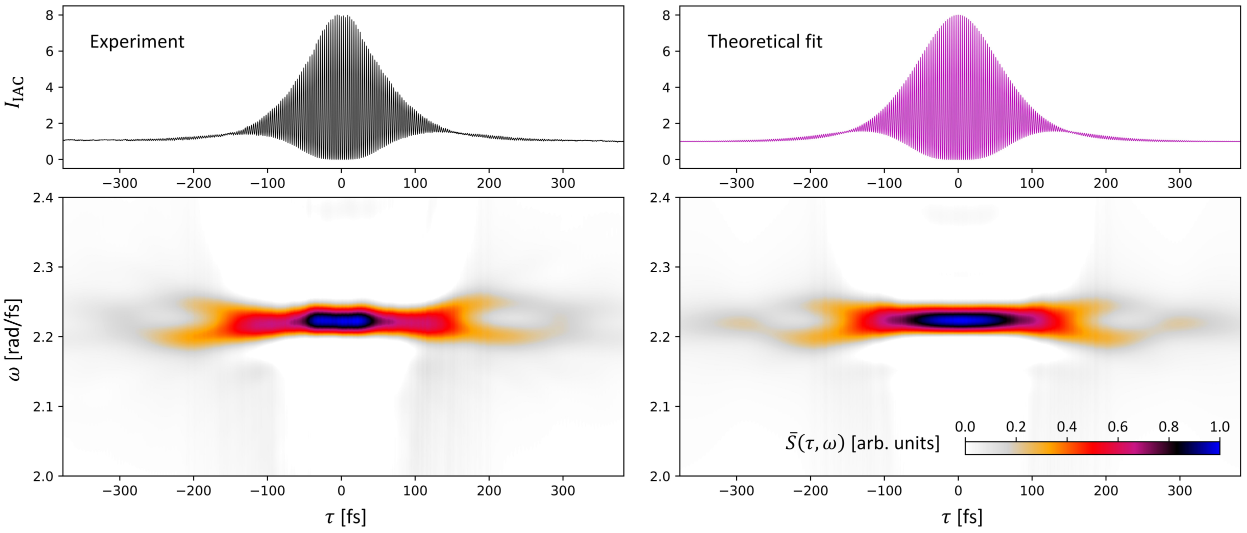

Figure S18: Experimental IAC curve (top left) and its spectrogram (bottom left), together with their theoretical fits (top right and bottom right, respectively), for the metasurface with L = 290 nm and Λ = 560 nm, under the incident light polarized parallel to the long axis of the nanoparticles. Fitted parameters: $\eta = 0.5823$, $\Omega_1 = 2.215$ rad/fs ($\lambda_{\text{res}} \approx 850.4$ nm), $\Gamma_1 = 0.011$ rad/fs (Q factor $= 101$), $\Omega_2 = 2.2341$ rad/fs ($\lambda_{\text{res}} \approx 843.1$ nm), $\Gamma_2 = 0.016797$ rad/fs (Q factor $= 67$), and $f_1/f_2 = 0.317$.

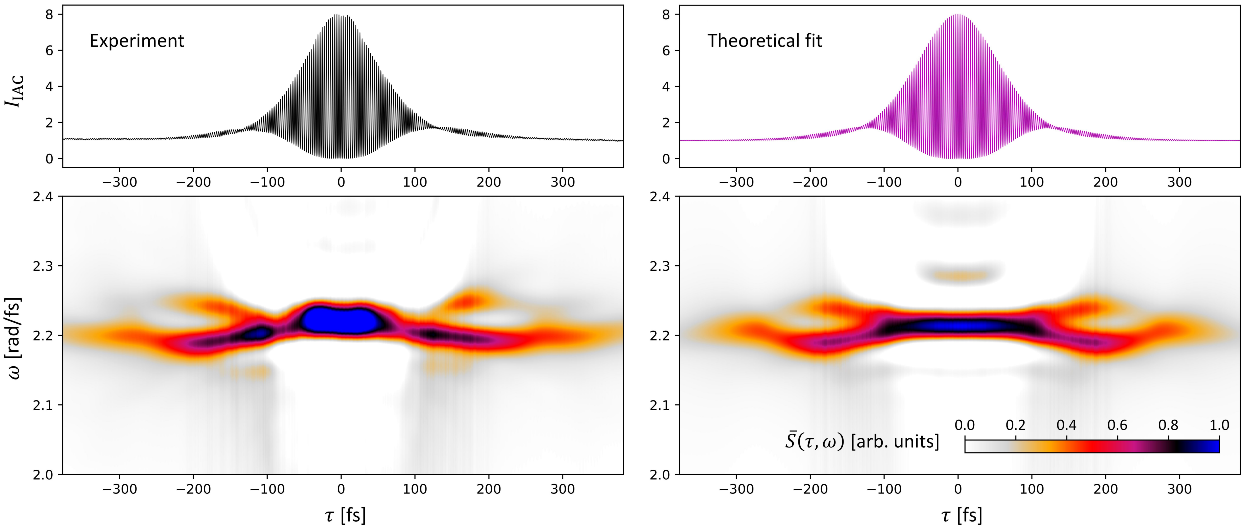

Figure S19: Experimental IAC curve (top left) and its spectrogram (bottom left), together with their theoretical fits (top right and bottom right, respectively), for the metasurface with L = 320 nm and Λ = 560 nm, under the incident light polarized parallel to the long axis of the nanoparticles. Fitted parameters: $\eta = 0.5927$, $\Omega_1 = 2.2055$ rad/fs ($\lambda_{res} \approx 854.1$ nm), $\Gamma_1 = 0.0105$ rad/fs (Q factor ≈ 105), $\Omega_2 = 2.2289$ rad/fs ($\lambda_{res} \approx 845.1$ nm), $\Gamma_2 = 0.0215$ rad/fs (Q factor ≈ 52), and $f_1/f_2 = 0.2563$.

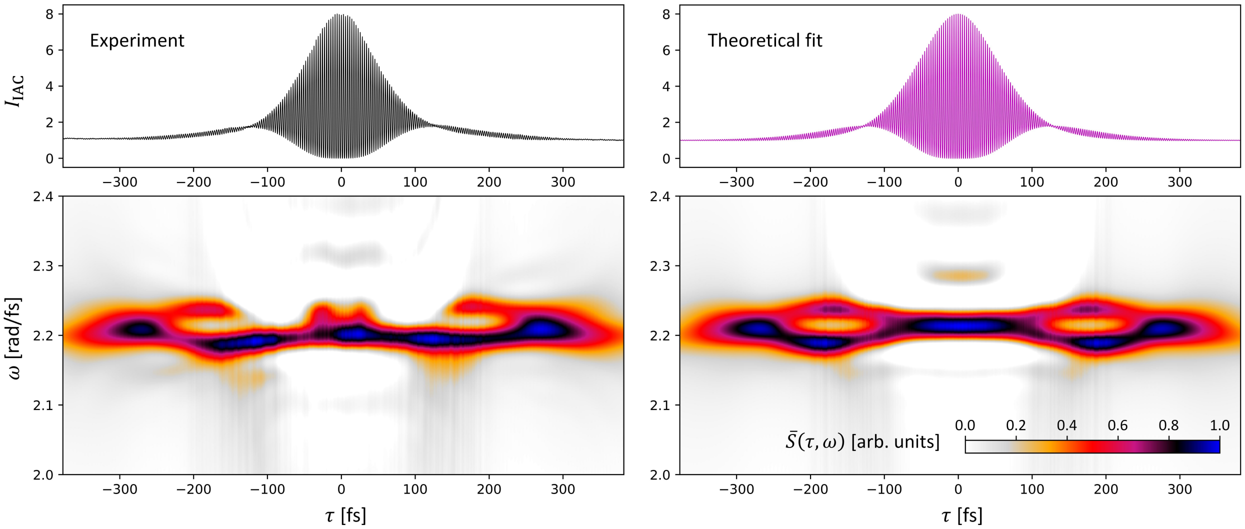

Figure S20: Experimental IAC curve (top left) and its spectrogram (bottom left), together with their theoretical fits (top right and bottom right, respectively), for the metasurface with L = 380 nm and Λ = 560 nm, under the incident light polarized parallel to the long axis of the nanoparticles. Fitted parameters: $\eta = 0.4664$, $\Omega_1 = 2.2055$ rad/fs ($\lambda_{res} \approx 854.1$ nm), $\Gamma_1 = 0.0087$ rad/fs (Q factor ≈ 127), $\Omega_2 = 2.2287$ rad/fs ($\lambda_{res} \approx 845.2$ nm), $\Gamma_2 = 0.014873$ rad/fs (Q factor ≈ 75), and $f_1/f_2 = 0.4068$.
